# Fatherhood and wage inequality in Britain, Finland, and Germany

**DOI:** 10.1111/jomf.12792

**Published:** 2021-08-11

**Authors:** Rossella Icardi, Anna Erika Hägglund, Mariña Fernández‐Salgado

**Affiliations:** ^1^ Department of Social and Policy Sciences University of Bath Bath UK; ^2^ Department of Social Research University of Turku Turku Finland; ^3^ Population Research Institute Family Federation of Finland Helsinki Finland; ^4^ Departamento de Economía Universidad de Alcalá Alcalá de Henares Spain

**Keywords:** fatherhood, inequality, wages

## Abstract

**Objective:**

This study investigates whether and how fatherhood shapes the wage distribution in Britain, Finland, and Germany.

**Background:**

Existing research debates whether fatherhood is associated with greater wages. However, it remains unclear whether the association between fatherhood and wages varies along the wage distribution as well as institutional contexts. To explore this, we compare three countries that differ in their wage bargaining institutions and family policies.

**Method:**

We use unconditional quantile regression on longitudinal data from the 1995 to 2016 waves of the Finnish Linked Employer Employee data, German Socio‐Economic Panel, and UK Longitudinal Household Study. To control for selection into fatherhood, we combine quantile regressions with fixed effects techniques.

**Results:**

Results show little evidence of substantial fatherhood wage effects along men's wage distribution. In all countries, fathers' higher wages at the median and top of the wage distribution are mostly accounted for by selection, but fatherhood shifts the bottom part of the distribution to the left particularly in the UK.

**Conclusions:**

The extent to which having a child affects men's wages across the wage distribution is similar across three diverse policy contexts. Yet, differences across the wage distribution are larger in the UK. We argue this may be linked to its higher level of inequality typical of liberal labour markets.

## INTRODUCTION

Parenthood stratifies men and women. While mothers' earnings penalties are well documented (Budig & England, 2001; Harkness & Waldfogel, 2003, Waldfogel, 1997, Gangl & Ziefle, 2009; Pal & Waldfogel, 2014; Single‐Rushton and Waldfogel, Sigle‐Rushton & Waldfogel, 2007), a growing number of studies show that fathers often earn more than childless men—the so‐called fatherhood wage premium (Budig & England, 2001; Glauber, 2008; Hodges & Budig, 2010; Killewald, 2013; Koslowski, 2011; Lundberg & Rose, 2000, 2002; Petersen, Penner, & Høgnes, 2014). Yet, the fatherhood wage effect is contested. First, studies document substantial cross‐national variation in fatherhood wage effects at the mean, with some countries displaying more pronounced and others little differences between fathers and childless men (Boeckmann & Budig, 2013; Koslowski, 2011). Second, recent studies have called into question whether the association between having children and wages is causal. Instead, they suggest that men transitioning into fatherhood experience steeper wage growth compared to men who remain childless. Fathers' higher wages would, thus, reflect positive selection on wage growth (Loghran and Zissimopolous, 2009; Ludwig & Brüderl, 2018; Mari, 2019).

To date, the association between fatherhood and wages has mostly been investigated at the mean. Existing North American studies point, however, to substantive group differences in fathers' wage advantages, as the magnitude differs among groups of men by factors associated with earnings level, such as educational attainment and occupations (Cooke & Fuller, 2018; Glauber, 2008; Hodges & Budig, 2010; Killewald, 2013). Similarly, the association between fatherhood and wages seems to differ across men's wage distribution, and existing research shows premia among higher earning men (Cooke, 2014; Glauber, 2018) and penalties for low earning ones (Cooke, 2014). Although these studies suggest that fatherhood contributes to wage inequalities among men (Cooke, 2014; Hodges & Budig, 2010), they do not sufficiently account for selection.

Systematic cross‐national comparisons of the association between fatherhood and wages are also rare (for exceptions, see Boeckman et al. Boeckmann & Budig, 2013; Koslowski, 2011; Mari, 2019). In addition, existing studies documenting variation in fathers' wage advantage across the wage distribution are based on liberal labour markets (Cooke, 2014; Glauber, 2018). Thus, it remains unclear whether distributional variations might be attenuated by the institutional context. The degree to which earnings vary between groups in a country is often related to the degree of labour market regulation (Blau & Kahn, 2003; Mandel & Semyonov, 2005), with literature drawing a distinction between liberal and coordinated market economies (Hall & Soskice, 2001). So far, however, research has not explored whether centralized wage structures also mitigate wage inequality stemming from fatherhood.

This study aims to fill this void by exploring whether and how fatherhood shapes the wage distribution in Finland, Germany and the United Kingdom. It contributes to the growing literature on the impact of children on men's labour market trajectories in two ways. First, it systematically compares whether and how fatherhood shapes the wage distribution across three diverse policy contexts. Second, by using longitudinal data, it explores how potential differences in wage trajectories among men may affect the shape of fatherhood premiums comparatively across the wage distribution. By incorporating these advances, this study offers a comprehensive analysis of how fatherhood effects emerge and how they shape the wage distribution of men across differing national contexts. It addresses the following research questions: Does the association between fatherhood and wages vary across the earnings distribution? If so, are distributional variations smaller in coordinated labour market economies with centralized wage structures?

The selected countries show different degrees of labour market coordination; moreover, they have distinct approaches to family policies. Although Germany and Finland continue to be characterized by a highly coordinated system of pay bargaining and relatively low levels of wage inequality, they differ in their policy support for dual earning/dual caring arrangements. The UK, in contrast, has no major industry‐wide agreements, and its meager family policies distinguishes it from the other two (OECD, 1996). Empirically, we use the 1995–2016 waves of high‐quality British, Finnish, and German national panel data to estimate unconditional quantile regressions with controls for individual fixed effects and wage growth profiles, allowing us to compare fatherhood estimates at different quantiles of men's unconditional earnings distribution.

## BACKGROUND

### Fatherhood effects across countries and the wage distribution

The question as to whether men benefit from fatherhood has generated a growing body of research. Several studies show that fathers often earn more than childless men (Koslowski, 2011; Lundberg & Rose, 2000, 2002; Petersen et al., 2014); yet, the magnitude of effects varies greatly across countries and by estimation strategy. For instance, research on the US tends to detect an average net effect of approximately 4% utilizing fixed effects estimators (Hodges & Budig, 2010), although the wage gain seems to be restricted to married (Killewald, 2013; Lundberg & Rose, 2000), biological and residential fathers (Killewald, 2013). More recent papers on the US, however, question these findings and argue, instead, that fathers' higher wages reflect steeper wage trajectories of fathers relative to childless men. Positive wage effects disappear once models adjust for these (Killewald & Lundberg, 2017; Loughran & Zissimopoulos, 2009; Ludwig & Brüderl, 2018).

Results on Europe, in turn, are mixed, and indicate country differences in how fatherhood shapes men's wages. For example, the fixed‐effects estimations of Koslowski (2011) detect some variation across 12 European countries, ranging from no difference in Finland to 9% and 11% higher wages among fathers in Germany vis‐à‐vis Denmark. Based on fixed‐effects models, Pollmann‐Schult (2011) establishes an average wage effect of approximately 2% for two children in West Germany, while Mari (2019) suggests that positive wage effects among German men reflect selection into fatherhood on wage trajectories. Bardasi and Taylor (2008) also highlight that British men do not receive a significant wage premium, and it diminishes further when controlling for time invariant characteristics; a conclusion supported by Mari (2019).

Investigations at the mean may hide important variations across the wage distribution and, thus, offer misleading results in the analysis of inequalities between groups (Bernhardt et al., 1995). In fact, existing studies show that the association between fatherhood and wages vary by level of education and occupation (Cooke & Fuller, 2018; Hodges & Budig, 2010), marital status (Glauber, 2008; Percheski & Wildeman, 2008), race (Glauber, 2008), and earnings level (Cooke, 2014; Glauber, 2018). Yet, these studies have mainly explored liberal labour markets, and most importantly, not taken selection sufficiently into consideration. For instance, Hodges and Budig (2010) find that the net fatherhood bonus in the US is greater for white men in professorial and managerial positions; a conclusion supported by Cooke and Fuller (2018) for Canada, but only net of establishment controls. So far, a restricted number of studies have also shown that the association between fatherhood and wages varies across earnings levels. Drawing on pooled data from the Current Population Survey, Glauber (2018) concludes that high‐earning men in the US receive a larger fatherhood wage premium than low‐ or middle‐earning men. The results of Cooke (2014), using the cross‐sectional Luxembourg Income Study, also align with this finding: net fatherhood premium increases as men's earnings increase in Australia, Britain and the U.S. Premiums at the top in all three countries are contrasted with significant wage losses among low earning men, although explanations for wage penalties remain unclear (Cooke, 2014; Mari, 2019).

To date, empirical evidence on variation in the association between fatherhood and wages is limited. First, distributional analyses on fatherhood wage effects have not utilized individual‐level panel data. Hence, the extent to which selection on time invariant characteristics or wage trajectories affect the shape of fatherhood premiums across the wage distribution remains unexplored. Second, studies on differences in fatherhood wage effects across the wage distribution have only focused on liberal labour markets (Cooke, 2014; Glauber, 2018) where wage inequality is generally higher and group differences larger (Blau & Kahn, 2003; Wallerstein, 1999). Thus, we still know little about the stratifying effect of fatherhood in countries with a more compressed wage structure.

### Theoretical explanations

Wage premiums for fathers are often assumed to result from three different mechanisms: treatment, employer discrimination, or selection (Petersen, Penner, & Høgnes, 2011). According to the treatment hypothesis, becoming a father induces men to change their behavior, for example, by paying more attention to work or by working harder. Fatherhood wage premiums would then reflect fathers' increased productivity (Lundberg & Rose, 2000). The second explanation relates to positive employer discrimination, which occurs if employers perceive fathers as more stable and reliable than childless men and reward these traits (Coltrane, 2004; Correll, Benard, Paik, & I., 2007; Hodges & Budig, 2010; Ridgeway & Correll, 2004). Measuring discrimination with survey data is difficult, but experimental studies offer some insights. For example, Correll et al. (2007) find that US undergraduate students evaluating job applications perceive fathers more favorably than childless men. Yet, call back rates among men remained unaffected by parental status. Similarly, experimental studies on Germany (Hipp, 2020) and Sweden (Bygren, Erlandsson, & Gähler, 2017) do not find support for positive discrimination of fathers in call backs.

Finally, positive selection, rather than behavioral changes, could account for the premium if men who become parents differ from non‐parents on characteristics valued by employers (Coltrane, 2004). Previous literature has detected such differences in both observed and stable unobserved characteristics such as work commitment and sense of responsibility (Hodges & Budig, 2010). At the same time, studies show that selection into parenthood varies across countries. For instance, while fathers are positively selected in Finland based on education (Nisén, Martikainen, Myrskylä, & Silventoinen, 2018) and employment trajectories (Jalovaara & Miettinen, 2013), the opposite holds true in the US (Hodges & Budig, 2010; Lundberg & Rose, 2000, 2002). A more recent debate, however, suggests that selection into fatherhood (and, closely related, marriage) also operates through wage profiles, in that fathers' wages grow steeper than those of childless men already prior to childbirth (or marriage). Hence, these studies ascertain that family formation occurs in a stage in the life when men's wages increase (Killewald & Lundberg, 2017; Loughran & Zissimopoulos, 2009; Ludwig & Brüderl, 2018; Mari, 2019).

These individual‐level mechanisms may operate differently across the earnings spectrum. Drawing on the treatment hypothesis, the economic payoff for increased productivity varies across groups of men. Greater productivity and job attention are especially expected from professionals in high paying occupations (Williams, Blair‐Loy, & Berdahl, 2013), and men at the top are also penalized the most when spending greater time doing routine housework (Cooke & Hook, 2018). This suggests that increasing efforts is more lucrative at the top, thus leading to larger benefits for high earning fathers. An increase in work effort could, however, also profit men at the bottom of the wage distribution. Highly educated (and high earning) couples usually espouse a gender egalitarian ideology and divide paid and unpaid work more equally, whereas the division of work is more likely to follow gender‐specific patterns when both partners have low educational levels (e.g., Sullivan, 2010). Hence, low earning couples might be more likely to adopt specialization. This suggests higher wage gains for low earners.

In terms of discrimination, the importance of responsibility and stability might vary according to the level of occupation (Magnusson, 2010). Highly prestigious and highly paid occupations are more likely to include high demands on “loyalty” to the organization and constant availability, including working overtime or taking part in organizational arrangements outside regular hours. Such work characteristics are of importance for wage growth (Gustafson, 2006; Magnusson & Nermo, 2017; Presser & Hermsen, 1996). This suggests that fathers at the top of the wage distribution are likely to gain a higher premium. Turning to selection, a study on class differences in parenting shows that particularly working‐class men perceive fatherhood as an obligation to provide for the family (Williams et al., 2013), suggesting that the positive wage effect might be more pronounced among low‐wage men. Similarly, it may also be more important for low earning men to have greater earnings potential and steeper wage trajectories when they become fathers. To date, however, there is no evidence on how selection on wage growth may vary among men with different earnings levels.

Taken together, the outlined mechanisms indicate that fatherhood affects wages in a different way along the wage distribution, although the direction is not clear. Specifically, if we assume that specialization benefits high earning men, or fathers' wage premia stem from discrimination, we should expect fatherhood effects to increase along the wage distribution and be higher at the top (H1a); conversely, if fatherhood effects reflect selection or if specialization is more pronounced among low‐wage men, effects should decrease along the wage distribution (H1b). If the three outlined mechanisms occur simultaneously, they might cancel each other out; in this event, we will not find evidence of distributional differences (H1c).

In all, identifying how these individual‐level mechanisms shape fatherhood across the wage distribution is challenging using survey data. Instead, we argue that differences in wages among men mirror broader labour market inequalities (Cooke & Fuller, 2018) and are, thus, connected to a country's institutional context.

### Variations across the wage distribution in Finland, Germany, and the UK

Existing research has mainly attributed cross‐national differences between fathers and childless men to family policies and cultural attitudes towards the division of household chores and childcare (Boeckmann & Budig, 2013; Bünning & Pollman‐Schult, 2016; Mari, 2019). Fathers' responsibilities as economic providers might differ by policy support to maternal employment and paternal caregiving. In contexts where male breadwinning is culturally reinforced (Townsend, 2002), fathers might enhance their work effort, or, anticipate this responsibility already prior to childbirth, leading to a stronger selection into fatherhood. In contrast, financial responsibility is lower in contexts where men and women share breadwinning and caregiving. Thus, if fatherhood wage effects foremost reflect breadwinning regimes, we would expect fatherhood effects to be larger in countries where male‐breadwinning prevails than in countries emphasizing dual‐earner and dual‐carer arrangements.

Yet, the extent to which fatherhood stratifies men may also be linked to the structure of wage‐setting institutions (Boeckmann & Budig, 2013). A substantial number of studies confirm that wage‐setting institutions affect the level of wage inequality (Blau & Kahn, 1996, 1999, 2003; Mandel & Semyonov, 2005; Wallerstein, 1999). All else equal, countries with relatively large differentials tend to have large pay gaps (Blau & Kahn, 2003). Earlier literature distinguishes between two types of economic models: liberal and coordinated market economies (Hall & Soskice, 2001). As compared to liberal economies, coordinated economies tend to be characterized by a higher degree of regulation, and a more comprehensive coverage of collective agreements. In general, heavily unionized economies with centralized bargaining systems have the lowest overall wage dispersion (Blau & Kahn, 1996). Previous studies have shown that wage differences among fathers are associated with interfirm wage differentials and establishment sorting (Cooke & Fuller, 2018). It is likely that centralized systems reducing wage variation across industries and firms also lower wage differentials stemming from fatherhood. Thus, if differences in fatherhood effects within countries reflect the overall level of wage dispersion, variations in these across the wage distribution should be less pronounced in coordinated market economies than in liberal ones.

The selected three countries present different combinations of both work‐family policies and degree of labour market coordination. The UK and Germany historically represent male breadwinning regimes, but differ in wage dispersion (OECD, 2018). While the UK offers low supports for families and maternal employment (Ray et al., 2010), the dominant German strategy has been to support a stay‐at‐home parent or one‐and‐a‐half earner arrangements by means of long child‐rearing leaves, part‐time early childhood care, and family taxation (Drasch, 2013). In 2007, Germany introduced a Nordic‐style parental leave scheme, with the aim to increase maternal employment and paternal care giving. In specific, the 2007 reform replaced the flat‐rate and partly means‐tested benefit, which could be obtained for 24 months, by an earnings‐related scheme of 12 months (Drasch, 2013). This new scheme also incentives couples to share parental leave for at least 2 months (“partner months”). In addition, availability of publicly subsidized childcare has increased continuously since the mid‐2000, although regional variation is pronounced (Zoch & Hondralis, 2017). Finally, Finland, together with the Nordic countries, promotes a dual‐earner/dual‐carer model (Haataja & Nyberg, 2006), in which extensive parental leave entitlements are coupled with publicly subsidized childcare (Eerola, Lammi‐Taskula, O'Brien, Hietamäki, & Räikkönen, 2019).

Turning to labour market coordination, the British collective bargaining system has always been decentralized to a considerable extent and wage bargaining is mostly un‐coordinated or takes place at the firm‐level (Cirillo, Sostero, & Tamagni, 2018). This has contributed to a high level of wage inequality in the country (Mutari & Figart, 2001). When collective agreements occur, they do not establish legally binding norms or contain contractual obligations (Visser, 2013). Overall, in the UK collective bargaining coverage is 27.5% (OECD, 2019). Germany presents a more centralized bargaining system with 57.8% bargaining coverage in 2015 (OECD, 2019), and wages are predominantly set at the sector or industry level (OECD, 2004). Although the degree of centralisation has guaranteed that the distribution of wages in Germany remained stable for a long time (Freeman & Katz, 1995), the country has seen a wide decline of collective bargaining agreements in the mid‐2000s, contributing to the rise in wage inequality (Felbermayr, Hauptmann, & Schmerer, 2014). In Finland income inequality is lower than in many OECD countries (OECD, 2019). While the country used to have a very high degree of centralization and co‐ordination between union and employers federations (OECD, 2006), major changes in the early 2000s contributed to a wider wage dispersion especially for white‐collar manufacturing workers and for those ranking high in the wage distribution (Asplund & Böckerman, 2008). Nevertheless, wage setting institutions continue to be more centralized compared to Britain and Germany, with a continuously high coverage for collective bargaining (89.3% in 2015; OECD, 2019).

These institutional differences are reflected in varying levels of wage inequality in each country, as evident in the 90/10 percentile ratio. This ratio is commonly used as a measure of wage inequality as it captures the gap between the high and low incomes; hence, the larger the ratio, the larger the wage dispersion. In 2019, it was lowest in Finland (3.0), larger in Germany (3.7) and largest in Britain (4.2; OECD, 2019) indicating that, for instance, in the latter top earners have a 4.2 times larger income than bottom ones.

Taken together, both family policies and labour market coordination could shape fatherhood wage effects in two distinct ways. First, if fatherhood wage effects reflect breadwinning regimes, we expect fatherhood to increase wages especially in contexts where male breadwinning is culturally reinforced. Thus, fathers' wage advantage should be more pronounced in the UK and Germany than in Finland (H2a). Second, liberal labour markets are characterized by overall higher levels of wage inequality and tend to have larger pay gaps. Thus, we expect the dispersion in fatherhood effects to be the largest in the UK, somewhat smaller in Germany and smallest in Finland (H2b).

## DATA AND METHODS

To analyze the wage impact of fatherhood in the three countries, we select the 1995–2016 waves of the German Socio‐Economic Panel (GSOEP; Gerstorf & Schupp, 2016; Wagner, Frick, & Schupp, 2007), the British Household Panel Survey merged with the UK Household Longitudinal Study (UKHLS; University of Essex, 2018), and a 1/3 random sample from the Finnish Linked Employee‐Employer Data (FLEED) merged with Structure of Earnings Statistics (OSF, 2018). The analytical sample includes 20 to 45 year old men who are either partnered (married or cohabiting) or who form a partnership during the observation window, and have at least two wage observations during the panel. We focus on partnered individuals to ensure that fatherhood wage effects do not reflect selection into partnership. Since the transition to partnership and to parenthood tend to occur within a limited time window, restricting the panel to years when men are already partnered also limits the observed wage trajectories. Therefore, we include the observations prior to partnership formation. Men are followed until they either leave the survey or when the number of children in the household decreases (e.g., because they move out or because they reach the age of 16, or 18 in Finland). Self‐employed, full‐time students, and person years with no valid wage information are excluded. In Finland, we also exclude individuals without a Finnish citizenship, as the number of missing values in the educational information for foreign degrees is high. German public sector employees receive a monthly family bonus; hence, they are excluded from the analysis (see Pollmann‐Schult, 2011). The same form of compensation does not exist in Britain or in Finland; hence, we do not exclude public sector workers in these countries but include a control variable (sensitivity checks for private sector only in the UK and Finland available in Tables S3). These selection criteria and the exclusion of all observation with missing information on any of the independent variables yield an analytic sample of 2919 British men (18,455 person‐years), 8443 German men (40,466 person‐years), and 203,382 Finnish men (1,518,750 person‐years). Table 1 summarizes all sample restrictions and relative sample sizes.

**TABLE 1:** Summary of sample restrictions and relative sample sizes

	Finland		Germany		UK	
	N person‐years	%	N person‐years	%	N person‐years	%
All men aged 20 to 45	6,683,422	100	104,233	100	47,052	100
In employment	4,315,474	0.65	72,791	0.70	34,642	0.74
Finnish citizenship (Finland only)	4,171,243	0.62				
Not missing	2,155,312	0.32	69,957	0.68	25,624	0.54
Partnered	1,785,266	0.27	58,068	0.56	21,355	0.45
Right censored	1,556,987	0.23	51,645	0.50	19,213	0.41
Private sector (Germany only)			42,352	0.41		
Observed for at least two waves	1,518,750	0.23	40,466	0.39	18,455	0.39

### Variables

The dependent variable is the natural logarithm of hourly wages (Hodges & Budig, 2010; Petersen, 1989). For Finland, the hourly wage stems from the Structure of Earnings Statistics, and is calculated by dividing the total gross earnings obtained in the reference month and the total number of paid working hours in the same month. For Germany and the UK, hourly wages are derived from gross monthly labour income (including paid overtime). This monthly income is divided by the amount of actual weekly working hours in the GSOEP and by expected weekly working hours in the BHPS, and multiplied by 4.33 (the approximate number of weeks in a month). Hours of paid overtime are excluded in the UK due to the large number of missing values. For all countries, wage observations above the 99th percentile were trimmed to reduce the influence of outliers on our estimates. Wage measures are adjusted using the 2014 Consumer Price Index.

The main independent variable, fatherhood, is measured as a time‐varying, discrete variable capturing the number of residential children (0, 1, 2, 3 and more children) under the age of 15 (18 for Finland), who live in the household with the fathers, as the salience of fatherhood is likely to diminish for non‐residential fathers (Knoester & Eggebeen, 2006). Commitment to fatherhood is lower for non‐residential fathers, because men's understanding of fatherhood and involvement with children are often tied to their relationship with the child's mother (Tach, Mincy and Edin, 2010).

Control variables include a Mincerian proxy for labour market experience (age minus years of education minus the 6 years prior to the start of compulsory schooling), its square term, education, and partnership status. Following Schneider (2016), the set of educational categories reflects the unique structure of each national education system. For Britain, we distinguish between GCSE (lower secondary) or below, A‐levels (upper secondary), and a university degree or higher. For Germany, we separate between no qualification or lower secondary, lower secondary with vocational qualification (referent), higher upper secondary (Abitur) with vocational qualification, and tertiary level qualification. For Finland, the distinction is between primary education (including missing, no distinction available between the two categories), vocational secondary (referent), general secondary (high school), lower tertiary, and upper tertiary. We distinguish between legally married and cohabiting including their years before partnering (referent). We also control for occupations (managers and professionals; technicians, clerks and service workers; and elementary and machine workers; based on 1988 International Standard Classification of Occupations) and for public sector, to account for differences across employment characteristics. Finally, region and year dummies are included to control for macro level trends.

### Analytical strategy

To assess the impact of fatherhood on the wage distribution of partnered men, we draw on a quantile regression technique. Two alternative techniques allow estimating the impact of explanatory variables on different parts of the earnings distribution, the conditional and unconditional approach. We avoid the conditional quantile estimator used in some studies (Albrecht, et al. 2015; Korpi, et al., 2013), because it is sensitive to which covariates are included in the model (Koenker, 2005). For example, including control variables for married individuals and having a university degree provides estimates across the wage distribution of married university graduates, not effects among all workers. Unconditional quantile regressions, by contrast, focus on the unconditional quantile of an individual, which is his/her earnings quantile in the overall earnings distribution, abstracting from observed and unobserved characteristics. Given our interest in assessing the impact of fatherhood on the unconditional male wage distribution, we follow previous studies (Cooke, 2014; England, Bearak, Budig, & Hodges, 2016; Glauber, 2018; Killewald & Bearak, 2014), and use Firpo, Fortin, and Lemiuex's (2009) unconditional quantile regression (UQR). The first step in estimating UQR is to calculate the recentered influence function (RIF) of the quantiles of the log of hourly wages. In a second step, the newly generated RIF is treated as the dependent variable in a pooled Ordinary Least Squares regression, which is regressed on the number of children in the household, and a set of covariates. A prerequisite for interpreting distributional effects as individual effects is that increases in the number of children do not alter a man's rank in the wage distribution (rank invariance; see Gregg, Macmillan, & Vittori, 2018). To avoid such assumption we interpret effects as shifts in the distribution.

We estimate unconditional quantile regressions at the 20^th^, 50^th^ and 80^th^ quantile of men's wage distribution with bootstrapped standard errors using 500 repetitions for Germany and the UK (Mooney and Duval, 1993). Model 1 assesses gross fatherhood effects accounting only for Mincerian labour market experience, region, and time. To take observed and time‐invariant unobserved heterogeneity at different points of the wage distribution into consideration (see Hodges & Budig, 2010; Lundberg & Rose, 2000), Model 2 includes controls for education, partnership status, occupations and the distinction between public and private sector (the latter only for Finland and the UK), as well as individual fixed effects. When estimating UQR and FE models with panel data, person‐years at each quantile should align with individual respondents in the wage distribution across years (England et al., 2016; Maroto, 2018). Our analyses reveal that most individuals remained in a wage percentile that was similar to their average percentile over time, necessary for FE‐UQR to be meaningful (England et al., 2016; see Figures S1a–c). To correct for potential differences in wage trajectories between fathers and childless men, Model 3 estimates a fixed effect model with group level slopes (FEGS). In specific, we include an interaction term between labour market experience and a dichotomous variable indicating whether a man ever experiences parenthood in the observation (“ever parent”) or remains childless.

### Methodological challenges

Recent literature has questioned whether fixed effects models are the most suitable estimation strategy to measure fatherhood wage effect, suggesting that fixed effects models are biased if men were more likely to have children when their wage trajectories are steeper (see e.g., Ludwig & Brüderl, 2018). Loughran and Zissimopoulos (2009) dealt with this limitation by estimating fixed effect models on first differences at the mean and found no effect for childbearing in the US. More recently, Ludwig and Brüderl (2018) analyzed the marriage premium at the mean in the US by estimating fixed effects models with individual‐specific constants and slopes (FEIS) and found neither a marriage nor a fatherhood premium for men. Similarly, Mari (2019) did not detect any wage bonus consequent to fatherhood in Germany and the UK, once models controlled for individual‐specific slopes (FEIS), except for modest premiums among older cohorts. None of the approaches suggested above can be easily applied to our analysis. First, estimating first differences using quantile regression is problematic, because the wage growth distribution does not correspond to the wage distribution. Second, in addition to being “data hungry”, the FEIS model provides a conservative test of the effect of fatherhood. In fact, by modeling trajectories at the individual level, the FEIS model absorbs part of the treatment effect if the effect changes over time. This could result in a downward bias in the parameter estimates (Meer & West, 2016). Therefore, instead of controlling for individual wage slopes (FEIS), we take differences in wage growth between never‐fathers and men eventually transitioning into fatherhood into consideration by including group‐specific slopes (FEGS). This latter model controls for heterogeneity in wage growth between (potential) fathers and never fathers. This estimation strategy is preferred over FEIS as it allows controlling for differences in wage growth without being too onerous on the data. In addition, it can be applied in combination with quantile regressions.

## RESULTS

Table S1 shows the distribution the dependent and independent variables in person‐years separately by fatherhood status and wage quantiles in Finland, Germany and the UK. Overall, average hourly wages were significantly larger for fathers in all countries. In the UK, fathers' average hourly wages were 13% larger than non‐fathers' wages, whereas the premium in Germany and Finland was 10 and 15%, respectively. Wage differentials at similar points in the wage distribution ranged from 1.5 to 3% in the UK. In Germany and Finland wage differences at the middle of the distribution were small, but there was a 1 and 2% wage gain for fathers at the bottom, respectively, and a 6 and 4% at the top. Although fathers in all countries had on average two children, patters across the distribution differed. In the UK, high and low earning fathers had a larger number of children than fathers with earnings closer to the median. High earning fathers in Germany and Finland, in turn, had more children than low earning fathers. To fully understand how these factors shape the association between fatherhood and men's earnings, we turn to the multivariate analyses.

### Fatherhood and wages across the wage distribution

Table 2 presents the association between fatherhood and wages along the wage distribution for each country and each of the three models (full models available in Tables S2). We began by reviewing the associations between having children and wages net of experience, region, and economic cycle (Model 1). Consistent with the descriptive statistics, fathers seemed to earn more than their childless counterparts in all quantiles in Germany and Finland, while differences in the UK were statistically significant only at the top. Hence, in all three countries having children shifted the wage distribution to the right. The selected countries displayed similar dispersions of fatherhood wage effects although they differed in terms of wage coordination and family policy regimes. Overall, the shape of effects corroborated Hypothesis 1a, according to which fatherhood effects should be larger for high earning men; yet, these models did not exhaustively account for selection and country differences therein.

**TABLE 2:** UQR coefficients of number of children on men's hourly wages in Finland, Germany, and the UK

Models	Finland			Germany			UK		
	20th q	50th q	80th q	20th q	50th q	80th q	20th q	50th q	80th q
Model 1: Gross	0.03***	0.05***	0.07***	0.01***	0.02***	0.05***	−0.01	0.01	0.02***
	(0.00)	(0.00)	(0.00)	(0.00)	(0.00)	(0.00)	(0.00)	(0.00)	(0.01)
Model 2: + FE	−0.01***	0.00	0.02***	−0.01	0.00	0.01	−0.04***	0.00	0.03***
	(0.00)	(0.00)	(0.00)	(0.01)	(0.01)	(0.01)	(0.01)	(0.01)	(0.01)
Model 3: + FEGS	−0.01***	0.00	0.01***	−0.01	0.00	0.01	−0.04***	0.00	0.02
	(0.00)	(0.00)	(0.00)	(0.01)	(0.01)	(0.01)	(0.01)	(0.01)	(0.02)
Person‐years	1,518,750	1,518,750	1,518,750	40,466	40,466	40,466	18,445	18,445	18,445

*Note*: *p < .1; **p < .05; ***p < .01. M1 controls for labour market experience, region, and time dummies; M2 adds controls for marital status, educational level, occupations, and individual fixed effects; M3 adds an interaction term between becoming a father and labour market experience.

Next, we examined how selection on observed and time‐invariant unobserved characteristics affected wages of fathers. To this end, we introduced controls for educational background, marital status, occupations, and public sector, as well as stable unobserved characteristics via the inclusion of individual fixed effects (Model 2). First, the positive association between the number of children and wages diminished in all countries across the entire wage distribution, with the only exception of the 80^th^ quantile in the UK. For the UK and Finland, Model 2 confirmed that the association between fatherhood and wages varies along the wage distribution, also when utilizing longitudinal data. In Germany, however, we found no evidence of fatherhood wage bonuses across the entire wage spectrum, despite the country's male breadwinning legacy. While this result partly contrasted previous empirical evidence (e.g., Koslowski, 2011), it is worth noticing that several studies on fatherhood effects in Germany did not exclude public sector employees (Cooke, 2014; Koslowski, 2011) who, in certain years, received a monthly family bonus. This may inflate the size of the fatherhood premium (Pollmann‐Schult, 2011).

Overall, the results pointed to positive selection into fatherhood. The decrease in fathers' wage advantage was most pronounced at the 20^th^ quantile, suggesting that particularly low‐wage men displayed time‐constant characteristics attractive to employers and women alike. In fact, in line with our expectations and previous cross‐sectional research on liberal labour markets (Cooke, 2014; Glauber, 2018), our results pointed to a negative wage effect at the 20^th^ quantile; this held not only in the UK, but also in Finland. Turning to the top, positive selection on observable and time invariant characteristics accounted for all premia in Germany and for a large share of Finnish fathers' higher wages; yet, a wage benefit remained. In contrast, we noticed a slight increase in the estimate at the 80^th^ quantile in the UK. This indicated that men at the top of the wage distribution in the UK negatively select into fatherhood, confirming patterns detected in the US (Hodges & Budig, 2010; Lundberg & Rose, 2000).

Once models adjust for differences in observable and stable unobservable characteristics, the dispersion of fatherhood wage effects seemed to be wider in the UK than in Finland (and Germany). In specific, fatherhood seemed to increase wage inequality among men in these countries. It is worth noting that estimates at the median approached zero in all models and across all three countries, corroborating studies that established no difference between fathers and childless men at the mean (Bardasi & Taylor, 2008; Koslowski, 2011; Ludwig & Brüderl, 2018; Mari, 2019).

To examine whether the fatherhood bonus is attributable to fathers' steeper wage trajectories, we controlled for wage slopes of men transitioning to fatherhood in the observation window (ever‐parent) and those who remain childless (Model 3). Results confirmed that the steepness of wage trajectories is a relevant factor for understanding wage differences between fathers and childless men; however, its relevance varied across the wage distribution. In Finland and the UK, the premia at the top indeed reflected differences in wage profiles, as the size of wage advantages decreased. This was somewhat unexpected in Finland, where we assumed that lower levels of wage inequality and stronger policy support for dual earner/dual carer arrangements should have mitigated the importance of wage growth among men. In contrast, fatherhood continued to shift the bottom part of the distribution to the left particularly in the UK, indicating that low‐wage fathers did not display different wage profiles than childless, low‐earning men. In Germany we did not find any additional changes once differences in slopes were accounted for. It is important to note that effect sizes in Germany were similar to Finland; however, because of smaller standard errors, they are significant only in the latter country. The difference in standard errors between the two countries was likely due to their differences in sample sizes.

While shifts in the distribution between fathers and childless men were mostly not significant at 5% level, it was still worth noting that patterns of dispersion aligned with our expectations. The difference in the association between fatherhood and wages between the top and the bottom quantiles shown in Table 3 was considerably larger in Britain (0.06) than in Finland (0.02) and Germany (0.02). Dispersion of fatherhood wage effects seemed to be larger in contexts where wage inequality is higher. This finding was in line with our expectation of larger distributional variation in liberal economies, where the overall level of wage inequality is higher (H2b). The results did not indicate that fathers' wage patterns would vary systematically by the degree of male‐ breadwinning (H2a). Put differently, fatherhood shifted the wage distribution similarly in Finland and Germany, although the countries are typically categorized as different family regime types.

**TABLE 3:** Interquantile difference of UQR fatherhood coefficients in Finland and UK

	Finland	Germany	UK
Overall (q80‐q20)	0.02***	0.02	0.06***
	(0.00)	(0.02)	(0.01)

*Note*: *p < .1; **p < .05; ***p < .01. Difference between UQR + FEGS coefficients (from Model 3); between q80 and q20.

Overall, our results provided new insights into how fatherhood affects men's wages. First, our systematic comparison of three policy contexts revealed both similarities and country differences in how fatherhood shapes wage inequality among men. In line with our expectations, we detected greater dispersion in effects in liberal UK than in Finland and Germany. Controlling for differences in wage trajectories between fathers and never‐fathers accounted for differences among high‐earning men in all countries apart from Finland, where a small premium remained. At the median and the top, our results aligned with findings of Ludwig and Brüderl (2018) and Mari (2019). Selection operates both *through wage levels*, sufficiently accounting for wage benefits at the median and at the bottom, and *earnings trajectories*, important at the top. Men tend to have children at a stage in their life characterized by a complex transition to adulthood, and men with higher earnings and earnings potential tend to select into fatherhood.

Once we accounted for selection, our results also showed that the association between the number of children and wages was negative among low‐wage fathers particularly in the UK, and to a smaller extent in Finland. Although theoretically unexpected, negative coefficients at the bottom could indicate that low‐wage men experience a slower wage growth once they have children. For instance, as Cooke and Fuller (2018) showed for Canada, less skilled fathers sort into lower wage firms when changing jobs. Under current economic and workplace circumstances, fulfilling material demands was not easy for working‐class fathers. Recent qualitative research suggests that job loss and job instability are a primary source of stress for these fathers (Roy & Dyson, 2010). Hence, they might avoid job changes, because of their fear of experiencing downward mobility or unemployment. Moreover, working class mothers also tend to work long hours to support the family (Scott, London, & Hurst, 2005). Men's chances to devote more effort to work may, thus, be hindered by their wives' working arrangements. This, in turn, could explain their lower wage growth.

### Sensitivity analyses

We have performed a set of additional sensitivity analyses. First, to assure comparability with Germany, we presented models only for private sector workers in Britain and Finland (Tables S3a,b). Results showed that, in the UK, fathers in the private sector experienced a slightly larger penalty at the bottom of the distribution whereas results remained unaltered at the top of the wage distribution. In Finland, the effects changed only marginally. Second, we also assessed how inequality levels within countries affected fatherhood wage effects, as variation in one country context over time should follow the same patterns as variation across countries. To this end, we estimated models including an interaction between the number of children and the standardized Gini index (Tables S4a–c); the latter is a commonly used measure for income inequality. For the UK and Finland, results aligned with the expected pattern: the level of inequality further expanded the wage distribution. In detail, in both countries penalties at the bottom of the wage distribution grew, when inequality increased. In the UK, inequality did not affect men at the top, whereas in Finland, premia at the top grew with increasing inequality. No effect was found in Germany. Third, we explored whether results for Germany varied before and after 2007, to investigate the potential consequence of the family reform on fatherhood estimates (Table S5). Results of this test showed a consistent pattern before and after the fatherhood reform, with the only difference of slightly larger coefficients at the top of the wage distribution after 2007.

## CONCLUSION

This study contributes to the growing literature on the impact of having children on men's wages by exploring whether fatherhood wage effects vary across the wage distribution in the UK, Finland, and Germany, three European countries characterized by different economic structures and varying degrees of wage inequality. As opposed to previous distributional analyses on fatherhood wage effects, conducted in liberal labour markets and with cross‐sectional data (e.g., Cooke, 2014; Glauber, 2018), this work draws on individual‐level longitudinal panel data. Thus, we can take selection more exhaustively into consideration and assess whether it operates differently across the diverse policy contexts and the wage spectrum.

In all, our results confirm that a cross‐nationally comparative, distributional approach provides further insights into the study of fatherhood wage effects. Having children seems to shift the wage distribution similarly across the three diverse policy contexts, in that we observe larger estimates at the top compared to the bottom of the distribution. Yet, in line with previous research (Bardasi & Taylor, 2008; Ludwig & Brüderl, 2018; Mari, 2019), we find little evidence of fatherhood wage effects at the median in any of the three selected countries, once we adjust for observed and unobserved differences between fathers and childless men. We also cannot detect substantial wage bonuses at the top. In specific, Finland is the only of our selected countries that display a small premium (1%) at the top coupled with a small penalty at the bottom (‐1%). Compared to the other countries, UK, displays the largest penalties for low earning men. This indicates that fatherhood shifts the wage distribution more strongly towards left. These patterns would go undetected by an estimation at the mean.

Fatherhood premiums are often assumed to result from three mechanisms—household specialization, employer discrimination, and selection into fatherhood. We argued that they could operate differently across the earnings spectrum, leading to varying effects at different quantiles of the wage distribution. The methodological approach and data used in this work did not allow us to explicitly test them. Nevertheless, our results provide additional evidence that positive fatherhood effects described in existing literature are primarily a result of selection, thus questioning the role of specialization and employer discrimination (see also Ludwig & Brüderl, 2018; Mari, 2019). In specific, the findings shed light on how selection plays out across the wage distribution and countries: men select into fatherhood both *through wage levels*, which sufficiently account for wage benefits at the median, and *earnings trajectories*, which explain premia at the top. Interestingly, selection does not differ substantially across the diverse policy contexts. For instance, family and social transfers are generally more generous in Finland than in the UK; yet, the transition to fatherhood is highly selective in both countries (see also Jalovaara, Andersson, & Miettinen, 2021).

Once we account for selection, we detect a negative association between having children and wages at the bottom in the UK; a finding consistent with previous research on liberal labour markets (Cooke, 2014). While we do not expect low‐wage men to be penalized by fatherhood, these negative associations could reflect low‐wage men's slower wage growth once they enter parenthood. Decreasing wage growth of working class fathers may be linked to their lower possibilities to change job, given their increased responsibilities as family providers (Cooke & Fuller, 2018). Correspondingly, they might live in dual‐earning households, where spouses' work schedules hamper their possibilities to show more work devotion (Scott et al., 2005; Williams, 2010). Our findings encourage future research to explore both theoretically and empirically the consequences of family formation among low‐wage men in high‐inequality contexts.

Also, we expected liberal labour markets to display higher levels of wage inequality stemming from fatherhood. Our results support this hypothesis. The countries differ in the variation across the wage distribution, with the interquantile difference being larger in the UK compared to Finland and Germany. The variation in fatherhood effects across the wage distribution seems, therefore, to mirror the overall level of wage dispersion when contrasting different labour market economies. Turning to family regimes, we assumed that fatherhood effects would be larger in countries where male‐breadwinning prevails and fathers' responsibilities as economic providers are more pronounced. Our results show that fatherhood shifts the wage distribution in the UK, but not in Germany, although both countries display male breadwinning legacies. While the 2007 reform in Germany introduced a Nordic‐style parental leave scheme with two partner months, studies do not establish wage disadvantages among men taking parental leave (Bünning, 2016). Thus, family regimes, which have been widely used to categorize women's wage patterns cross‐nationally (e.g., Gangl & Ziefle, 2009), and men's working hours (Bünning & Pollman‐Schult, 2016), may not be an optimal framework to explain men's wages in response to fatherhood.

This study contributes to the debate about the sources of inequality among men and the overall level of inequality within countries. While scholars conventionally have contrasted the negative effect of children on mothers' earnings with fathers' wage advantages, our findings add to the evidence that the “fatherhood premium” predominantly is the result of selection. Yet, our results also show that selection mechanisms vary across wage distribution. In addition, fatherhood seems to increase disadvantage among low‐earning men in high inequality contexts. Thus, we encourage scholars to assess selection dynamics in men's fertility patterns in greater detail, systematically comparing mechanisms both across different socio‐political contexts as well as groups of men. Understanding *who* becomes a father, at which *time point* this transition takes place, and whether these patterns *vary across groups*, requires also taking women's and couples' position in the wage structure into consideration. Thus, analyzing fatherhood and its relation to the earnings distribution continues to be an important task for future research.

## Supporting information


**TABLE S1** Descriptive statistics for the United Kingdom, Finland and Germany.
**TABLE S2a**. UQR coefficients for number of children across men's hourly wage distribution, Finland.
**TABLE S2c**. UQR coefficients for number of children across men's hourly wage distribution, United Kingdom.
**TABLE S3a**. UQR coefficients for number of children in household across men's hourly wage distribution. Private sector only, Finland.
**TABLE S3b**. UQR coefficients for number of children in household across men's hourly wage distribution. Private sector only, United Kingdom.
**FIGURE S1a**. Difference in percentile rank in wages from mean ranking, Finland.
**FIGURE S1b**. Difference in percentile rank in wages from mean ranking, Germany.
**FIGURE S1c**. Difference in percentile rank in wages from mean ranking, United Kingdom.
**TABLE S4a**. UQR coefficients for number of children across men's hourly wage distribution, controlling for inequality levels. Finland.
**TABLE S4b**. UQR coefficients for number of children across men's hourly wage distribution, controlling for inequality levels. Germany.
**TABLE S4c**. UQR coefficients for number of children across men's hourly wage distribution, controlling for inequality levels. United Kingdom.
**TABLE S5**. UQR coefficients for number of children across men's hourly wage distribution, pre and post 2007 reform. Germany.
