# Supplementary material for: Fatherhood and wage inequality in Britain, Finland, and Germany
Source: J Marriage Fam. 2021 Aug 11;84(1):273–90. doi: 10.1111/jomf.12792 (PMC9292225; doi:10.1111/jomf.12792)
Supplement: Supplementary file 1 — TABLE S1 Descriptive statistics for the United Kingdom, Finland and Germany. TABLE S2a. UQR coefficients for number of children across men's hourly wage distribution, Finland. TABLE S2c. UQR coefficients for number of children across men's hourly wage distribution, United Kingdom. TABLE S3a. UQR coefficients for number of children in household across men's hourly wage distribution. Private sector only, Finland. TABLE S3b. UQR coefficients for number of children in household across men's hourly wage distribution. Private sector only, United Kingdom. FIGURE S1a. Difference in percentile rank in wages from mean ranking, Finland. FIGURE S1b. Difference in percentile rank in wages from mean ranking, Germany. FIGURE S1c. Difference in percentile rank in wages from mean ranking, United Kingdom. TABLE S4a. UQR coefficients for number of children across men's hourly wage distribution, controlling for inequality levels. Finland. TABLE S4b. UQR coefficients for number of children across men's hourly wage distribution, controlling for inequality levels. Germany. TABLE S4c. UQR coefficients for number of children across men's hourly wage distribution, controlling for inequality levels. United Kingdom. TABLE S5. UQR coefficients for number of children across men's hourly wage distribution, pre and post 2007 reform. Germany. [file JOMF-84-273-s001.docx]

**Online Supplement**

**Table S1. Descriptive statistics for the United Kingdom, Finland and Germany. Online Only.**

|  | All men | | <20th | | 20th-50th | | 50th-80th | | >80th | |
| --- | --- | --- | --- | --- | --- | --- | --- | --- | --- | --- |
|  | Non fathers | Fathers | Non fathers | Fathers | Non fathers | Fathers | Non fathers | Fathers | Non fathers | Fathers |
| **UK** |  |  |  |  |  |  |  |  |  |  |
| Hourly wage (£) | 14.26 | 16.18 | 7.49 | 7.63 | 11.37 | 11.55 | 16.52 | 16.79 | 26.45 | 27.43 |
| Nr children |  | 1.81 |  | 1.84 |  | 1.77 |  | 1.80 |  | 1.86 |
| Age | 31.50 | 36.23 | 29.04 | 33.68 | 30.93 | 35.46 | 32.37 | 36.79 | 34.90 | 38.29 |
| Experience(yrs) | 14.93 | 19.69 | 12.75 | 17.56 | 14.54 | 19.17 | 15.61 | 20.20 | 17.85 | 21.22 |
| Ever parent | 0.46 |  | 0.44 |  | 0.46 |  | 0.51 |  | 0.43 |  |
| GCSE or below | 0.29 | 0.35 | 0.43 | 0.57 | 0.34 | 0.45 | 0.22 | 0.32 | 0.12 | 0.12 |
| A levels | 0.32 | 0.29 | 0.37 | 0.31 | 0.38 | 0.36 | 0.29 | 0.30 | 0.22 | 0.19 |
| Tertiary education | 0.38 | 0.35 | 0.21 | 0.12 | 0.28 | 0.19 | 0.49 | 0.38 | 0.67 | 0.69 |
| Unmarried | 0.25 |  | 0.36 |  | 0.26 |  | 0.18 |  | 0.14 |  |
| Married | 0.37 | 0.82 | 0.26 | 0.69 | 0.35 | 0.77 | 0.43 | 0.86 | 0.50 | 0.92 |
| Cohabiting | 0.38 | 0.18 | 0.38 | 0.31 | 0.39 | 0.23 | 0.39 | 0.14 | 0.36 | 0.07 |
| **N** | 7538 | 10917 | 1836 | 1846 | 2388 | 3145 | 2121 | 3414 | 1193 | 2512 |
| **Finland** |  |  |  |  |  |  |  |  |  |  |
| Hourly wage (€) | 17.73 | 20.97 | 12.03 | 12.30 | 15.88 | 15.93 | 20.52 | 20.70 | 29.74 | 31.12 |
| Nr children |  | 1.95 |  | 1.86 |  | 1.92 |  | 1.95 |  | 2.02 |
| Age | 30.09 | 36.36 | 27.36 | 34.36 | 29.82 | 35.58 | 31.49 | 36.49 | 33.97 | 38.06 |
| Experience(yrs) | 10.74 | 17.02 | 8.78 | 16.40 | 10.91 | 17.36 | 11.62 | 17.14 | 12.77 | 16.83 |
| Ever parent | 0.52 |  | 0.51 |  | 0.52 |  | 0.54 |  | 0.52 |  |
| No secondary educ | 0.09 | 0.08 | 0.14 | 0.17 | 0.10 | 0.12 | 0.05 | 0.06 | 0.02 | 0.02 |
| High school diploma | 0.13 | 0.05 | 0.20 | 0.06 | 0.11 | 0.04 | 0.09 | 0.04 | 0.09 | 0.05 |
| Vocational upper secondary | 0.40 | 0.38 | 0.50 | 0.56 | 0.49 | 0.56 | 0.34 | 0.37 | 0.12 | 0.10 |
| Low tertiary | 0.23 | 0.30 | 0.13 | 0.15 | 0.23 | 0.23 | 0.31 | 0.37 | 0.29 | 0.35 |
| High tertiary | 0.15 | 0.20 | 0.03 | 0.06 | 0.07 | 0.05 | 0.21 | 0.15 | 0.47 | 0.48 |
| Unmarried | 0.42 |  | 0.50 |  | 0.43 |  | 0.37 |  | 0.30 |  |
| Married | 0.19 | 0.80 | 0.12 | 0.71 | 0.17 | 0.75 | 0.23 | 0.81 | 0.34 | 0.89 |
| Cohabiting | 0.39 | 0.20 | 0.38 | 0.29 | 0.40 | 0.25 | 0.40 | 0.19 | 0.36 | 0.11 |
| Private | 0.70 | 0.65 | 0.67 | 0.57 | 0.71 | 0.66 | 0.72 | 0.67 | 0.69 | 0.67 |
| Municipal/local | 0.12 | 0.16 | 0.17 | 0.27 | 0.11 | 0.17 | 0.08 | 0.11 | 0.13 | 0.15 |
| State | 0.18 | 0.19 | 0.17 | 0.15 | 0.18 | 0.17 | 0.20 | 0.22 | 0.18 | 0.19 |
| **N** | 670673 | 848077 | 190161 | 113461 | 219367 | 236279 | 179169 | 276505 | 81976 | 221832 |
| **Germany** |  |  |  |  |  |  |  |  |  |  |
| Hourly wage (€) | 15.23 | 16.85 | 7.75 | 7.84 | 12.52 | 12.49 | 17.70 | 17.75 | 27.16 | 28.81 |
| Nr children |  | 1.82 |  | 1.74 |  | 1.80 |  | 1.83 |  | 1.90 |
| Age | 32.69 | 36.65 | 30.61 | 35.17 | 31.60 | 35.70 | 33.79 | 36.94 | 35.76 | 38.66 |
| Experience(yrs) | 14.13 | 18.33 | 12.98 | 17.79 | 13.78 | 18.35 | 14.78 | 18.74 | 15.30 | 18.22 |
| Ever parent | 0.35 |  | 0.35 |  | 0.34 |  | 0.33 |  | 0.35 |  |
| No education, low secondary | 0.09 | 0.10 | 0.14 | 0.15 | 0.12 | 0.14 | 0.07 | 0.09 | 0.02 | 0.03 |
| Low secondary +voc | 0.56 | 0.58 | 0.68 | 0.69 | 0.63 | 0.67 | 0.51 | 0.60 | 0.34 | 0.32 |
| Abitur +vocational | 0.11 | 0.10 | 0.11 | 0.08 | 0.11 | 0.10 | 0.11 | 0.12 | 0.09 | 0.32 |
| Tertiary | 0.24 | 0.22 | 0.08 | 0.08 | 0.13 | 0.09 | 0.32 | 0.19 | 0.56 | 0.55 |
| Unmarried | 0.28 |  | 0.33 |  | 0.30 |  | 0.25 |  | 0.22 |  |
| Married | 0.38 | 0.91 | 0.32 | 0.82 | 0.36 | 0.90 | 0.41 | 0.93 | 0.47 | 0.94 |
| Cohabiting | 0.34 | 0.09 | 0.35 | 0.16 | 0.34 | 0.09 | 0.34 | 0.06 | 0.31 | 0.06 |
| **N** | 12132 | 28334 | 2754 | 5225 | 3855 | 8311 | 3652 | 8493 | 1851 | 6325 |

**Table S2a. UQR coefficients for number of children across men’s hourly wage distribution, Finland. Online Only.**

|  | M1: GROSS | | | | | | M2: FE | | | | | | M3: FEGS | | | | | |
| --- | --- | --- | --- | --- | --- | --- | --- | --- | --- | --- | --- | --- | --- | --- | --- | --- | --- | --- |
|  | Q20 |  | Q50 |  | Q80 |  | Q20 |  | Q50 |  | Q80 |  | Q20 |  | Q50 |  | Q80 |  |
|  | b/se |  | b/se |  | b/se |  | b/se |  | b/se |  | b/se |  | b/se |  | b/se |  | b/se |  |
| Nr children | 0.03 | *** | 0.05 | *** | 0.07 | *** | -0.01 | *** | 0.00 |  | 0.02 | *** | -0.01 | *** | 0.00 |  | 0.01 | *** |
|  | (0.00) |  | (0.00) |  | (0.00) |  | (0.00) |  | (0.00) |  | (0.00) |  | (0.00) |  | (0.00) |  | (0.00) |  |
| Ever parent x experience | | |  |  |  |  |  |  |  |  |  |  | 0.00 | *** | 0.00 |  | 0.01 | *** |
|  |  |  |  |  |  |  |  |  |  |  |  |  | (0.00) |  | (0.00) |  | (0.00) |  |
| Experience | 0.04 | *** | 0.05 | *** | 0.05 | *** | -0.05 | *** | 0.05 | *** | 0.10 | *** | -0.05 | *** | 0.05 | *** | 0.09 | *** |
|  | (0.00) |  | (0.00) |  | (0.00) |  | (0.01) |  | (0.00) |  | (0.00) |  | (0.00) |  | (0.00) |  | (0.00) |  |
| Experience sq | -0.00 | *** | -0.00 | *** | -0.00 | *** | -0.00 | *** | -0.00 | *** | -0.00 | *** | 0.00 | *** | 0.00 | *** | 0.00 | *** |
|  | (0.00) |  | (0.00) |  | (0.00) |  | (0.00) |  | (0.00) |  | (0.00) |  | (0.00) |  | (0.00) |  | (0.00) |  |
| Education | | | |  |  |  |  |  |  |  |  |  |  |  |  |  |  |  |
| Primary (or missing) | |  |  |  |  |  | -0.15 | *** | 0.02 | *** | 0.16 | *** | -0.15 | *** | 0.02 | *** | 0.15 | *** |
|  |  |  |  |  |  |  | (0.01) |  | (0.01) |  | (0.01) |  | (0.01) |  | (0.01) |  | (0.01) |  |
| Upper Secondary |  |  |  |  |  |  | -0.14 | *** | -0.03 | *** | 0.04 | *** | -0.14 | *** | -0.03 | *** | 0.03 | *** |
|  |  |  |  |  |  |  | (0.01) |  | (0.01) |  | (0.01) |  | (0.01) |  | (0.00) |  | (0.01) |  |
| Lower Tertiary |  |  |  |  |  |  | 0.07 | *** | 0.12 | *** | -0.02 | *** | 0.07 | *** | 0.12 | *** | -0.02 | *** |
|  |  |  |  |  |  |  | (0.00) |  | (0.00) |  | (0.01) |  | (0.00) |  | (0.00) |  | (0.01) |  |
| Upper Tertiary |  |  |  |  |  |  | 0.03 | *** | 0.21 | *** | 0.21 | *** | 0.03 | *** | 0.21 | *** | 0.21 | *** |
|  |  |  |  |  |  |  | (0.01) |  | (0.01) |  | (0.01) |  | (0.01) |  | (0.01) |  | (0.01) |  |
| Marital status | | |  |  |  |  |  |  |  |  |  |  |  |  |  |  |  |  |
| Married |  |  |  |  |  |  | 0.04 | *** | 0.03 | *** | -0.02 | *** | 0.04 | *** | 0.03 | *** | -0.02 | *** |
|  |  |  |  |  |  |  | (0.00) |  | (0.00) |  | (0.00) |  | (0.00) |  | (0.00) |  | (0.00) |  |
| Cohabiting |  |  |  |  |  |  | 0.03 | *** | 0.00 | ** | -0.04 | *** | 0.03 | *** | 0.00 | *** | -0.04 | *** |
|  |  |  |  |  |  |  | (0.00) |  | (0.00) |  | (0.00) |  | (0.00) |  | (0.00) |  | (0.00) |  |
| Sector | |  |  |  |  |  |  |  |  |  |  |  |  |  |  |  |  |  |
| Municipality |  |  |  |  |  |  | -0.06 | *** | -0.06 | *** | -0.06 | *** | -0.06 | *** | -0.06 | *** | -0.06 | *** |
|  |  |  |  |  |  |  | (0.00) |  | (0.00) |  | (0.00) |  | (0.00) |  | (0.00) |  | (0.00) |  |
| State |  |  |  |  |  |  | -0.00 |  | -0.05 | *** | -0.09 | *** | 0 |  | -0.05 | *** | -0.09 | *** |
|  |  |  |  |  |  |  | (0.00) |  | (0.00) |  | (0.00) |  | (0.00) |  | (0.00) |  | (0.00) |  |
| Occupation | | |  |  |  |  |  |  |  |  |  |  |  |  |  |  |  |  |
| Managers&Professionals | |  |  |  |  |  | 0.06 | *** | 0.10 | *** | 0.11 | *** | 0.06 | *** | 0.1 | *** | 0.11 | *** |
|  |  |  |  |  |  |  | (0.00) |  | (0.00) |  | (0.00) |  | (0.00) |  | (0.00) |  | (0.00) |  |
| Technicians&Service&Clerks |  |  |  |  |  |  | 0.04 | *** | 0.04 | *** | -0.06 | *** | 0.04 | *** | 0.04 | *** | -0.06 | *** |
|  |  |  |  |  |  |  | (0.00) |  | (0.00) |  | (0.00) |  | (0.00) |  | (0.00) |  | (0.00) |  |
| Constant | 2.41 | *** | 2.71 | *** | 3.10 | *** | 1.494 | *** | 2.76 | *** | 3.62 | *** | 1.49 | *** | 2.76 | *** | 3.64 | *** |
|  | (0.00) |  | (0.00) |  | (0.00) |  | (0.05) |  | (0.03) |  | (0.04) |  | (0.05) |  | (0.03) |  | (0.04) |  |
| R-squared | 0.13 |  | 0.14 |  | 0.11 |  | 0.20 |  | 0.24 |  | 0.18 |  | 0.20 |  | 0.24 |  | 0.18 |  |
| Observations | 1518750 |  | 1518750 |  | 1518750 |  | 1518750 |  | 1518750 |  | 1518750 |  | 1518750 |  | 1518750 |  | 1518750 |  |

Note: * 0.1 ** 0.05 *** 0.01. Models include also year and region dummies.

**Table S2b. UQR coefficients for number of children across men’s hourly wage distribution, Germany. Online Only.**

|  | M1: GROSS | | | | | | M2: FE | | | | | | M3: FEGS | | | | |  |
| --- | --- | --- | --- | --- | --- | --- | --- | --- | --- | --- | --- | --- | --- | --- | --- | --- | --- | --- |
|  | Q20 |  | Q50 |  | Q80 |  | Q20 |  | Q50 |  | Q80 |  | Q20 |  | Q50 |  | Q80 |  |
|  | b/se |  | b/se |  | b/se |  | b/se |  | b/se |  | b/se |  | b/se |  | b/se |  | b/se |  |
| Nr children | 0.01 | *** | 0.02 | *** | 0.05 | *** | -0.01 |  | 0.00 |  | 0.01 |  | -0.01 |  | 0.00 |  | 0.01 |  |
|  | (0.00) |  | (0.00) |  | (0.00) |  | (0.01) |  | (0.01) |  | (0.01) |  | (0.01) |  | (0.01) |  | (0.01) |  |
| Ever parent x experience |  |  |  |  |  |  |  |  |  |  |  |  | 0.01 | ** | 0.00 |  | 0.00 |  |
|  |  |  |  |  |  |  |  |  |  |  |  |  | (0.00) |  | (0.00) |  | (0.00) |  |
| Experience | 0.05 | *** | 0.05 | *** | 0.05 | *** | 0.04 | *** | 0.04 | *** | 0.02 | *** | 0.02 | *** | 0.03 | *** | 0.04 | *** |
|  | (0.00) |  | (0.00) |  | (0.00) |  | (0.01) |  | (0.01) |  | (0.01) |  | (0.01) |  | (0.00) |  | (0.01) |  |
| Experience sq | -0.00 | *** | -0.00 | *** | -0.00 | *** | -0.00 | *** | -0.00 | *** | -0.00 | *** | -0.00 | *** | -0.00 | *** | -0.00 | *** |
|  | (0.00) |  | (0.00) |  | (0.00) |  | (0.00) |  | (0.00) |  | (0.00) |  | (0.00) |  | (0.00) |  | (0.00) |  |
| East Germany | -0.56 | *** | -0.36 | *** | -0.28 | *** | -0.34 | *** | -0.10 | *** | 0.00 |  | -0.34 | *** | -0.11 | *** | 0.00 |  |
|  | (0.01) |  | (0.01) |  | (0.01) |  | (0.05) |  | (0.03) |  | (0.04) |  | (0.04) |  | (0.03) |  | (0.03) |  |
| No education, low secondary |  |  |  |  |  |  | -0.02 |  | 0.00 |  | 0.06 | ** | 0.03 |  | 0.06 | ** | 0.09 | *** |
|  |  |  |  |  |  |  | (0.03) |  | (0.02) |  | (0.02) |  | (0.04) |  | (0.03) |  | (0.03) |  |
| Abitur+voc |  |  |  |  |  |  | 0.06 | ** | 0.04 |  | 0.08 | ** | 0.01 |  | -0.02 |  | 0.00 |  |
|  |  |  |  |  |  |  | (0.03) |  | (0.02) |  | (0.03) |  | (0.05) |  | (0.04) |  | (0.05) |  |
| Tertiary |  |  |  |  |  |  | 0.32 | *** | 0.23 | *** | 0.24 | *** | 0.22 | *** | 0.12 | *** | 0.08 |  |
|  |  |  |  |  |  |  | (0.05) |  | (0.03) |  | (0.04) |  | (0.06) |  | (0.04) |  | (0.05) |  |
| Married |  |  |  |  |  |  | 0.08 | *** | 0.05 | *** | -0.00 |  | 0.09 | *** | 0.06 | *** | -0.00 |  |
|  |  |  |  |  |  |  | (0.02) |  | (0.01) |  | (0.02) |  | (0.02) |  | (0.01) |  | (0.02) |  |
| Cohabiting |  |  |  |  |  |  | 0.04 | *** | 0.02 | * | -0.01 |  | 0.05 |  | 0.02 |  | -0.01 |  |
|  |  |  |  |  |  |  | (0.02) |  | (0.01) |  | (0.01) |  | (0.02) |  | (0.01) |  | (0.01) |  |
| Managers&Professionals |  |  |  |  |  |  | -0.03 |  | 0.02 | * | 0.00 |  | -0.02 |  | 0.03 | ** | 0.01 |  |
|  |  |  |  |  |  |  | (0.02) |  | (0.01) |  | (0.02) |  | (0.02) |  | (0.02) |  | (0.02) |  |
| Technicians&Service&Clerks |  |  |  |  |  |  | -0.04 | ** | -0.00 |  | -0.04 | *** | -0.04 | ** | -0.00 | ** | -0.04 | *** |
|  |  |  |  |  |  |  | (0.02) |  | (0.01) |  | (0.01) |  | (0.08) |  | (0.05) |  | (0.05) |  |
| Constant | 2.03 | *** | 2.28 | *** | 2.56 | *** | 1.87 | *** | 2.14 | *** | 2.60 | *** | 2.05 | *** | 2.33 | *** | 2.64 | *** |
|  | (0.03) |  | (0.02) |  | (0.03) |  | (0.06) |  | (0.04) |  | (0.06) |  | (0.05) |  | (0.04) |  | (0.05) |  |
| R-squared | 0.12 |  | 0.10 |  | 0.05 |  | 0.03 |  | 0.05 |  | 0.05 |  | 0.03 |  | 0.05 |  | 0.05 |  |
| Observations | 40466 |  | 40466 |  | 40466 |  | 40466 |  | 40466 |  | 40466 |  | 40466 |  | 40466 |  | 40466 |  |

Note: * 0.1 ** 0.05 *** 0.01. Models include also year dummies.

**Table S2c. UQR coefficients for number of children across men’s hourly wage distribution, United Kingdom. Online Only.**

|  |  |  | M1: GROSS | | |  |  |  | M2: FE | |  |  | M3: FEGS | | | | | |
| --- | --- | --- | --- | --- | --- | --- | --- | --- | --- | --- | --- | --- | --- | --- | --- | --- | --- | --- |
|  | Q20 |  | Q50 |  | Q80 |  | Q20 |  | Q50 |  | Q80 |  | Q20 |  | Q50 |  | Q80 |  |
|  | b/se |  | b/se |  | b/se |  | b/se |  | b/se |  | b/se |  | b/se |  | b/se |  | b/se |  |
| Nr children | -0.01 |  | 0.01 |  | 0.02 | *** | -0.04 | *** | 0.00 |  | 0.03 | *** | -0.04 | *** | 0.00 |  | 0.02 |  |
|  | (0.00) |  | (0.00) |  | (0.01) |  | (0.01) |  | (0.01) |  | (0.01) |  | (0.01) |  | (0.01) |  | (0.02) |  |
| Ever parent x experience |  |  |  |  |  |  |  |  |  |  |  |  | 0.00 |  | 0.00 |  | 0.01 | *** |
|  |  |  |  |  |  |  |  |  |  |  |  |  | (0.00) |  | (0.00) |  | (0.01) |  |
| Experience | 0.09 | *** | 0.08 | *** | 0.06 | *** | 0.01 |  | 0.05 |  | 0.01 |  | 0.00 |  | 0.05 |  | 0.01 |  |
|  | (0.00) |  | (0.00) |  | (0.00) |  | (0.07) |  | (0.15) |  | (0.04) |  | (0.04) |  | (0.09) |  | (0.02) |  |
| Experience sq | -0.00 | *** | -0.00 | *** | -0.00 | *** | -0.00 | *** | -0.00 | *** | -0.00 | *** | -0.00 | *** | -0.00 | *** | -0.00 | *** |
|  | (0.00) |  | (0.00) |  | (0.00) |  | (0.00) |  | (0.00) |  | (0.00) |  | (0.00) |  | (0.00) |  | (0.00) |  |
| A-levels |  |  |  |  |  |  | 0.15 | *** | -0.08 | ** | -0.15 | *** | 0.15 | ** | -0.08 | * | -0.16 | *** |
|  |  |  |  |  |  |  | (0.05) |  | (0.04) |  | (0.05) |  | (0.08) |  | (0.06) |  | (0.07) |  |
| Tertiary |  |  |  |  |  |  | 0.12 | * | 0.03 |  | -0.08 |  | 0.12 | * | 0.03 |  | -0.09 |  |
|  |  |  |  |  |  |  | (0.06) |  | (0.06) |  | (0.07) |  | (0.08) |  | (0.08) |  | (0.10) |  |
| Married |  |  |  |  |  |  | 0.08 | *** | 0.05 | *** | -0.06 | ** | 0.08 | *** | 0.05 | ** | -0.05 | ** |
|  |  |  |  |  |  |  | (0.02) |  | (0.02) |  | (0.02) |  | (0.04) |  | (0.03) |  | (0.04) |  |
| Cohabiting |  |  |  |  |  |  | 0.09 | *** | 0.03 | * | -0.05 | ** | 0.09 | *** | 0.03 | * | -0.04 | ** |
|  |  |  |  |  |  |  | (0.02) |  | (0.02) |  | (0.02) |  | (0.03) |  | (0.02) |  | (0.03) |  |
| Managers&Professionals |  |  |  |  |  |  | 0.02 |  | 0.07 | *** | 0.06 | *** | 0.02 |  | 0.07 | *** | 0.06 | *** |
|  |  |  |  |  |  |  | (0.02) |  | (0.01) |  | (0.02) |  | (0.03) |  | (0.02) |  | (0.02) |  |
| Technicians&Service&Clerks |  |  |  |  |  |  | -0.05 | ** | -0.00 |  | -0.00 |  | -0.05 | * | -0.00 |  | -0.00 |  |
|  |  |  |  |  |  |  | (0.02) |  | (0.01) |  | (0.02) |  | (0.03) |  | (0.02) |  | (0.02) |  |
| Private sector | -0.15 | *** | -0.10 | *** | 0.01 |  | -0.12 | *** | 0.02 |  | 0.10 | *** | -0.12 | *** | 0.02 |  | 0.10 | *** |
|  | (0.01) |  | (0.01) |  | (0.01) |  | (0.02) |  | (0.02) |  | (0.02) |  | (0.03) |  | (0.03) |  | (0.03) |  |
| Constant | 1.39 | *** | 1.83 | *** | 2.25 | *** | 1.97 | *** | 1.93 |  | 2.57 | *** | 1.97 | *** | 1.93 | ** | 2.57 | *** |
|  | (0.04) |  | (0.03) |  | (0.04) |  | (0.64) |  | (1.38) |  | (0.35) |  | (0.35) |  | (0.82) |  | (0.17) |  |
| R-Squared | 0.10 |  | 0.10 |  | 0.07 |  | 0.12 |  | 0.13 |  | 0.12 |  | 0.12 |  | 0.13 |  | 0.12 |  |
| Observations | 18455 |  | 18455 |  | 18455 |  | 18455 |  | 18455 |  | 18455 |  | 18455 |  | 18455 |  | 18455 |  |

Note: * 0.1 ** 0.05 *** 0.01. Models include also year and region dummies.

**Table S3a. UQR coefficients for number of children in household across men’s hourly wage distribution. Private sector only, Finland. Online Only.**

|  |  |  | M2: FE |  |  |  |  |  | M3: FEGS | |  |  |
| --- | --- | --- | --- | --- | --- | --- | --- | --- | --- | --- | --- | --- |
|  | Q20 |  | Q50 |  | Q80 |  | Q20 |  | Q50 |  | Q80 |  |
|  | b/se |  | b/se |  | b/se |  | b/se |  | b/se |  | b/se |  |
| Nr. of children | -0.01 | *** | 0.00 |  | 0.02 | *** | -0.01 | *** | 0.00 |  | 0.01 | *** |
|  | (0.00) |  | (0.00) |  | (0.00) |  | (0.00) |  | (0.00) |  | (0.00) |  |
| Ever parent x experience |  |  |  |  |  |  | 0.00 | *** | 0.00 | * | 0.01 | *** |
|  |  |  |  |  |  |  | (0.00) |  | (0.00) |  | (0.00) |  |
| Experience | -0.07 | *** | 0.03 | *** | 0.09 | *** | -0.06 | *** | 0.03 | *** | 0.09 | *** |
|  | (0.01) |  | (0.00) |  | (0.01) |  | (0.01) |  | (0.00) |  | (0.01) |  |
| Experience sq | -0.00 | *** | -0.00 | *** | -0.00 | *** | 0.00 | *** | 0.00 | *** | 0.00 | *** |
|  | (0.00) |  | (0.00) |  | (0.00) |  | (0.00) |  | (0.00) |  | (0.00) |  |
| Education level () |  |  |  |  |  |  |  |  |  |  |  |  |
| Primary (or missing) | -0.14 | *** | 0.02 | * | 0.14 | *** | -0.13 | *** | 0.02 | * | 0.14 | *** |
|  | (0.01) |  | (0.01) |  | (0.01) |  | (0.01) |  | (0.01) |  | (0.01) |  |
| Upper secondary | -0.09 | *** | -0.02 | *** | 0.00 |  | -0.09 | *** | -0.02 | *** | 0.00 |  |
|  | (0.01) |  | (0.01) |  | (0.01) |  | (0.01) |  | (0.01) |  | (0.01) |  |
| Lower tertiary | 0.09 | *** | 0.11 | *** | -0.02 | ** | 0.09 | *** | 0.11 | *** | -0.02 | ** |
|  | (0.01) |  | (0.01) |  | (0.01) |  | (0.01) |  | (0.01) |  | (0.01) |  |
| Higher tertiary | -0.01 |  | 0.22 | *** | 0.28 | *** | -0.01 |  | 0.22 | *** | 0.27 | *** |
|  | (0.01) |  | (0.01) |  | (0.01) |  | (0.01) |  | (0.01) |  | (0.01) |  |
| Married | 0.03 | *** | 0.02 | *** | -0.02 | *** | 0.03 | *** | 0.02 | *** | -0.02 | *** |
|  | (0.00) |  | (0.00) |  | (0.00) |  | (0.00) |  | (0.00) |  | (0.00) |  |
| Cohabiting | 0.03 | *** | 0.00 |  | -0.04 | *** | 0.03 | *** | 0.00 |  | -0.04 | *** |
|  | (0.00) |  | (0.00) |  | (0.00) |  | (0.00) |  | (0.00) |  | (0.00) |  |
| Managers&Professionals | 0.04 | *** | 0.09 | *** | 0.13 | *** | 0.04 | *** | 0.09 | *** | 0.13 | *** |
|  | (0.00) |  | (0.00) |  | (0.01) |  | (0.00) |  | (0.00) |  | (0.00) |  |
| Technicians&Service&Clerks | 0.05 | *** | 0.05 | *** | -0.07 | *** | 0.05 | *** | 0.05 | *** | -0.07 | *** |
|  | (0.00) |  | (0.00) |  | (0.00) |  | (0.00) |  | (0.00) |  | (0.00) |  |
| Constant | 1.26 | *** | 2.56 | *** | 3.60 | *** | 1.25 | *** | 2.56 | *** | 3.62 | *** |
|  | (0.06) |  | (0.04) |  | (0.06) |  | (0.06) |  | (0.04) |  | (0.05) |  |
| Observations | 1022580 |  | 102280 |  | 102280 |  | 1022580 |  | 1022580 |  | 1022580 |  |
| R-squared | 0.21 |  | 0.25 |  | 0.200 |  | 0.21 |  | 0.25 |  | 0.20 |  |

Note: * 0.1 ** 0.05 *** 0.01. Models include also year and region dummies.

**Table S3b. UQR coefficients for number of children in household across men’s hourly wage distribution. Private sector only, United Kingdom. Online Only.**

|  |  |  | M2: FE |  |  |  |  |  | M3: FEGS | |  |  |
| --- | --- | --- | --- | --- | --- | --- | --- | --- | --- | --- | --- | --- |
|  | Q20 |  | Q50 |  | Q80 |  | Q20 |  | Q50 |  | Q80 |  |
|  | b/se |  | b/se |  | b/se |  | b/se |  | b/se |  | b/se |  |
| Nr children | -0.05 | *** | -0.01 |  | 0.03 | ** | -0.05 | *** | -0.01 |  | 0.02 |  |
|  | (0.01) |  | (0.01) |  | (0.01) |  | (0.02) |  | (0.01) |  | (0.02) |  |
| Ever parent x Experience | | |  |  |  |  | 0.00 |  | 0.00 |  | 0.01 | *** |
|  |  |  |  |  |  |  | (0.01) |  | (0.00) |  | (0.01) |  |
| Experience | 0.01 |  | 0.02 |  | 0.01 |  | 0.01 |  | 0.01 |  | 0.00 |  |
|  | (0.07) |  | (0.10) |  | (0.03) |  | (0.04) |  | (0.07) |  | (0.02) |  |
| Experience sq | -0.00 | *** | -0.00 | *** | -0.00 | *** | -0.00 | *** | -0.00 | *** | -0.00 | *** |
|  | (0.00) |  | (0.00) |  | (0.00) |  | (0.00) |  | (0.00) |  | (0.00) |  |
| A-levels | 0.14 | ** | -0.07 |  | -0.20 | *** | 0.14 | ** | -0.07 |  | -0.21 | *** |
|  | (0.06) |  | (0.06) |  | (0.07) |  | (0.09) |  | (0.08) |  | (0.10) |  |
| Tertiary | 0.04 |  | 0.07 |  | -0.02 |  | 0.04 |  | 0.07 |  | -0.02 |  |
|  | (0.07) |  | (0.07) |  | (0.10) |  | (0.10) |  | (0.10) |  | (0.15) |  |
| Married | 0.06 | ** | 0.06 | *** | -0.04 |  | 0.06 | ** | 0.06 | ** | -0.03 |  |
|  | (0.03) |  | (0.02) |  | (0.03) |  | (0.04) |  | (0.04) |  | (0.05) |  |
| Cohabiting | 0.09 | *** | 0.03 |  | -0.04 |  | 0.10 | *** | 0.03 |  | -0.03 |  |
|  | (0.03) |  | (0.02) |  | (0.02) |  | (0.03) |  | (0.03) |  | (0.03) |  |
| Managers&Professional | 0.00 |  | 0.06 | *** | 0.08 | *** | 0.00 |  | 0.06 | *** | 0.09 | *** |
|  | (0.02) |  | (0.02) |  | (0.02) |  | (0.03) |  | (0.02) |  | (0.03) |  |
| Technicians&Service &Clerks | -0.05 | ** | 0.00 |  | -0.00 |  | -0.05 |  | 0.00 |  | 0.00 |  |
|  | (0.02) |  | (0.02) |  | (0.02) |  | (0.03) |  | (0.02) |  | (0.03) |  |
| Constant | 1.86 | *** | 2.23 | ** | 2.66 | *** | 1.86 | *** | 2.23 | *** | 2.67 | *** |
|  | (0.67) |  | (0.89) |  | (0.29) |  | (0.33) |  | (0.60) |  | (0.20) |  |
| R-Squared | 0.11 |  | 0.12 |  | 0.11 |  | 0.11 |  | 0.12 |  | 0.11 |  |
| Observations | 14299 |  | 14299 |  | 14299 |  | 14299 |  | 14299 |  | 14299 |  |

Note: * 0.1 ** 0.05 *** 0.01. Models include also year dummies.

**Percentile rank in wages over time**

When estimating UQR and FE models with panel data, it is important to note that wage groups at each quantile refer to groups of person-years. These are defined by their similar net worth levels, which that do not necessarily align with individual persons. Because individuals, as opposed to person-years, are more intuitive unit of analysis (see England et al., 2016), we have explored percentile rank-invariance for individuals across years. Figures O2 provides estimates for the proportion of individuals whose percentile rank in the wage distribution at each data point (year) differed from their average percentile rank (across years) by 10, 25, and 33 percentage points. Across years, approximately 20-22% of individuals differed from their mean percentile rank by 33 percentage points or more, some 50% differed by at least 25 percentage points, and ca. 60% differed by 10 percentage points or greater. Movement across quantiles was similar when compared with the median rank, but less volatility was present with the median differences.

Taken together, these results show that most individuals remained in a wage percentile that was similar to their average percentile over time. Even though individuals’ wage levels changed across years, the percentile rank within the distribution was relatively stable for most respondents.

**FIGURE S1a. DIFFERENCE IN PERCENTILE RANK IN WAGES FROM MEAN RANKING, FINLAND. . Online Only.**


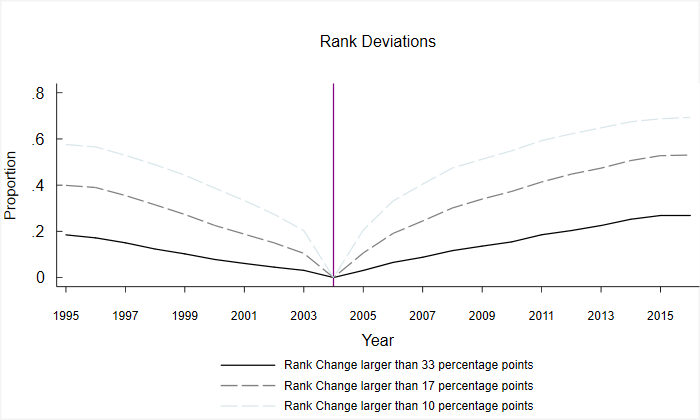


**FIGURE S1b. DIFFERENCE IN PERCENTILE RANK IN WAGES FROM MEAN RANKING, GERMANY. Online Only.**


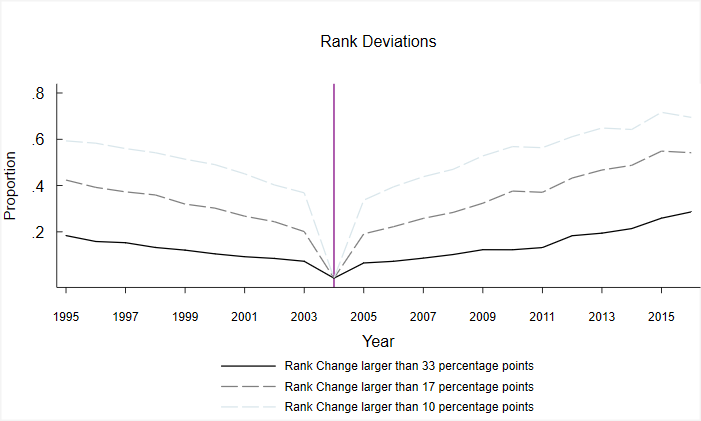


**FIGURE S1c. DIFFERENCE IN PERCENTILE RANK IN WAGES FROM MEAN RANKING, UNITED KINGDOM. Online Only.**


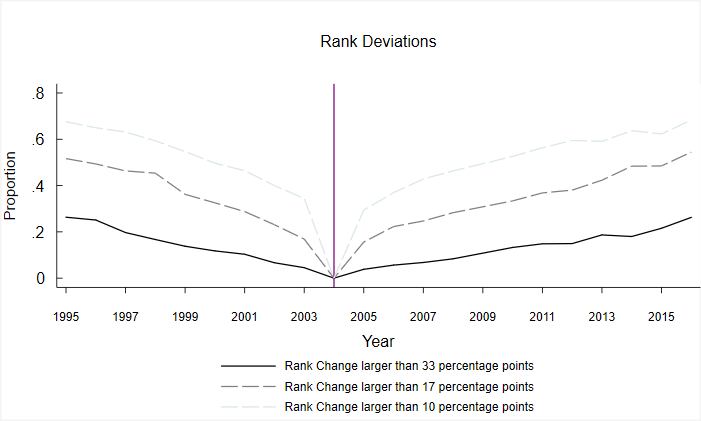


**Table S4a. UQR coefficients for number of children across men’s hourly wage distribution, controlling for inequality levels. Finland. Online Only.**

|  |  |  | M2: FE |  |  |  |  |  | M3: FEGS | |  |  |
| --- | --- | --- | --- | --- | --- | --- | --- | --- | --- | --- | --- | --- |
|  | Q20 |  | Q50 |  | Q80 |  | Q20 |  | Q50 |  | Q80 |  |
|  | b/se |  | b/se |  | b/se |  | b/se |  | b/se |  | b/se |  |
| Nr children | -0.01 | *** | 0.00 |  | 0.02 | *** | -0.01 | *** | 0.00 |  | 0.01 | *** |
|  | (0.00) |  | (0.00) |  | (0.00) |  | (0.00) |  | (0.00) |  | (0.00) |  |
| Gini (std) | 0.05 | *** | 0.01 | *** | -0.02 | *** | 0.05 | *** | 0.01 | *** | -0.02 | *** |
|  | (0.00) |  | (0.00) |  | (0.00) |  | (0.00) |  | (0.00) |  | (0.00) |  |
| Nr children*Gini (std) | -0.01 | *** | 0.00 |  | 0.01 | *** | -0.01 | *** | -0.00 |  | 0.01 | *** |
|  | (0.00) |  | (0.00) |  | (0.00) |  | (0.00) |  | (0.00) |  | (0.00) |  |
| Ever parent x experience |  |  |  |  |  |  | -0.00 | *** | 0.00 | *** | 0.01 | *** |
|  |  |  |  |  |  |  | (0.00) |  | (0.00) |  | (0.00) |  |
| Experience | 0.04 | *** | 0.04 | *** | 0.06 | *** | 0.04 | *** | 0.04 | *** | 0.05 | *** |
|  | (0.00) |  | (0.00) |  | (0.00) |  | (0.00) |  | (0.00) |  | (0.00) |  |
| Experience sq | -0.00 | *** | -0.00 | *** | -0.00 | *** | -0.00 | *** | -0.00 | *** | -0.00 | *** |
|  | (0.00) |  | (0.00) |  | (0.00) |  | (0.00) |  | (0.00) |  | (0.00) |  |
| Primary(missing) | -0.15 | *** | 0.02 | *** | 0.16 | *** | -0.15 | *** | 0.02 | *** | 0.15 | *** |
|  | (0.01) |  | (0.01) |  | (0.01) |  | (0.01) |  | (0.01) |  | (0.01) |  |
| Upper secondary | -0.14 | *** | -0.03 | *** | 0.04 | *** | -0.14 | *** | -0.03 | *** | 0.04 | *** |
|  | (0.01) |  | (0.00) |  | (0.01) |  | (0.01) |  | (0.00) |  | (0.01) |  |
| Lower Tertiary | 0.08 | *** | 0.12 | *** | -0.02 | *** | 0.08 | *** | 0.12 | *** | -0.02 | *** |
|  | (0.00) |  | (0.00) |  | (0.01) |  | (0.00) |  | (0.00) |  | (0.01) |  |
| Upper Tertiary | 0.05 | *** | 0.21 | *** | 0.20 | *** | 0.05 | *** | 0.21 | *** | 0.20 | *** |
|  | (0.01) |  | (0.01) |  | (0.01) |  | (0.01) |  | (0.01) |  | (0.01) |  |
| Married | 0.04 | *** | 0.03 | *** | -0.01 | *** | 0.04 | *** | 0.03 | *** | -0.02 | *** |
|  | (0.00) |  | (0.00) |  | (0.00) |  | (0.00) |  | (0.00) |  | (0.00) |  |
| Cohabiting | 0.03 | *** | 0.00 | ** | -0.04 | *** | 0.03 | *** | 0.00 | ** | -0.04 | *** |
|  | (0.00) |  | (0.00) |  | (0.00) |  | (0.00) |  | (0.00) |  | (0.00) |  |
| Municipality | -0.06 | *** | -0.06 | *** | -0.07 | *** | -0.06 | *** | -0.06 | *** | -0.07 | *** |
|  | (0.00) |  | (0.00) |  | (0.00) |  | (0.00) |  | (0.00) |  | (0.00) |  |
| State | 0.00 |  | -0.05 | *** | -0.09 | *** | 0.00 |  | -0.05 | *** | -0.09 | *** |
|  | (0.00) |  | (0.00) |  | (0.00) |  | (0.00) |  | (0.00) |  | (0.00) |  |
| Managers&Professionals | 0.06 | *** | 0.10 | *** | 0.11 | *** | 0.06 | *** | 0.10 | *** | 0.11 | *** |
|  | (0.00) |  | (0.00) |  | (0.00) |  | (0.00) |  | (0.00) |  | (0.00) |  |
| Technicians&Service& Clerks | 0.04 | *** | 0.04 | *** | -0.06 | *** | 0.04 | *** | 0.04 | *** | -0.06 | *** |
|  | (0.00) |  | (0.00) |  | (0.00) |  | (0.00) |  | (0.00) |  | (0.00) |  |
| GDP | -0.00 | *** | -0.00 | *** | -0.00 | *** | -0.00 | *** | -0.00 | *** | -0.00 | *** |
|  | (0.00) |  | (0.00) |  | (0.00) |  | (0.00) |  | (0.00) |  | (0.00) |  |
| Unemployment | -0.01 | *** | -0.01 | *** | -0.01 | *** | -0.01 | *** | -0.01 | *** | -0.01 | *** |
|  | (0.00) |  | (0.00) |  | (0.00) |  | (0.00) |  | (0.00) |  | (0.00) |  |
| Constant | 2.70 | *** | 2.92 | *** | 3.33 | *** | 2.70 | *** | 2.92 | *** | 3.33 | *** |
|  | (0.01) |  | (0.01) |  | (0.01) |  | (0.01) |  | (0.01) |  | (0.01) |  |
| R-squared | 0.20 |  | 0.24 |  | 0.18 |  | 0.20 |  | 0.24 |  | 0.18 |  |
| Observations | 1518750 |  | 1518750 |  | 1518750 |  | 1518750 |  | 1518750 |  | 1518750 |  |

Note: * 0.1 ** 0.05 *** 0.01. Models also include region dummies.

**Table S4b. UQR coefficients for number of children across men’s hourly wage distribution, controlling for inequality levels. Germany. Online Only.**

|  |  |  | M2: FE |  |  |  |  |  | M3: FEGS | |  |  |
| --- | --- | --- | --- | --- | --- | --- | --- | --- | --- | --- | --- | --- |
|  | Q20 |  | Q50 |  | Q80 |  | Q20 |  | Q50 |  | Q80 |  |
|  | b/se |  | b/se |  | b/se |  | b/se |  | b/se |  | b/se |  |
| Nr children | -0.01 |  | 0.00 |  | 0.01 |  | -0.01 |  | 0.01 |  | 0.01 |  |
|  | (0.01) |  | (0.01) |  | (0.01) |  | (0.01) |  | (0.01) |  | (0.01) |  |
| Gini (std) | -0.04 | ** | -0.01 |  | 0.02 |  | -0.04 | ** | -0.01 |  | 0.02 |  |
|  | (0.01) |  | (0.01) |  | (0.01) |  | (0.01) |  | (0.01) |  | (0.01) |  |
| Nr children*Gini (std) | 0.01 |  | -0.00 |  | -0.00 |  | 0.01 |  | -0.00 |  | -0.00 |  |
|  | (0.01) |  | (0.00) |  | (0.01) |  | (0.01) |  | (0.00) |  | (0.01) |  |
| Ever parent x experience | |  |  |  |  |  | 0.00 |  | -0.00 |  | -0.00 |  |
|  |  |  |  |  |  |  | (0.00) |  | (0.00) |  | (0.00) |  |
| Experience | 0.04 | *** | 0.05 | *** | 0.04 | *** | 0.04 | *** | 0.05 | *** | 0.04 | *** |
|  | (0.01) |  | (0.01) |  | (0.01) |  | (0.01) |  | (0.01) |  | (0.01) |  |
| Experience sq | -0.00 | *** | -0.00 | *** | -0.00 | *** | -0.00 | *** | -0.00 | *** | -0.00 | *** |
|  | (0.00) |  | (0.00) |  | (0.00) |  | (0.00) |  | (0.00) |  | (0.00) |  |
| No education, low secondary | -0.01 |  | -0.02 |  | 0.01 |  | -0.01 |  | -0.02 |  | 0.01 |  |
|  | (0.04) |  | (0.03) |  | (0.03) |  | (0.04) |  | (0.03) |  | (0.03) |  |
| Abitur+vocational | 0.06 |  | 0.05 |  | 0.06 |  | 0.06 |  | 0.05 |  | 0.06 |  |
|  | (0.05) |  | (0.04) |  | (0.05) |  | (0.05) |  | (0.04) |  | (0.05) |  |
| Tertiary | 0.30 | *** | 0.24 | *** | 0.19 | *** | 0.30 | *** | 0.24 | *** | 0.20 | *** |
|  | (0.07) |  | (0.05) |  | (0.07) |  | (0.07) |  | (0.05) |  | (0.07) |  |
| Married | 0.08 | *** | 0.04 | ** | -0.01 |  | 0.08 | *** | 0.04 | ** | -0.01 |  |
|  | (0.02) |  | (0.02) |  | (0.03) |  | (0.02) |  | (0.02) |  | (0.03) |  |
| Cohabiting | 0.04 | ** | 0.02 |  | -0.01 |  | 0.04 | ** | 0.02 |  | -0.01 |  |
|  | (0.02) |  | (0.01) |  | (0.02) |  | (0.02) |  | (0.01) |  | (0.02) |  |
| Managers&Professional | -0.03 |  | 0.03 |  | 0.01 |  | -0.03 |  | 0.03 |  | 0.01 |  |
|  | (0.02) |  | (0.02) |  | (0.02) |  | (0.02) |  | (0.02) |  | (0.02) |  |
| Technicians&Service &Clerks | -0.04 | * | -0.00 |  | -0.04 | ** | -0.04 | * | -0.00 |  | -0.04 | ** |
|  | (0.02) |  | (0.02) |  | (0.02) |  | (0.02) |  | (0.02) |  | (0.02) |  |
| East Germany | -0.34 | *** | -0.11 | ** | -0.00 |  | -0.34 | *** | -0.11 | ** | -0.00 |  |
|  | (0.08) |  | (0.05) |  | (0.05) |  | (0.08) |  | (0.05) |  | (0.05) |  |
| GDP | 0.00 |  | -0.00 |  | 0.00 | ** | 0.00 |  | -0.00 |  | 0.00 | ** |
|  | (0.00) |  | (0.00) |  | (0.00) |  | (0.00) |  | (0.00) |  | (0.00) |  |
| Unemployment rate | -0.00 |  | 0.01 | * | 0.01 | ** | -0.00 |  | 0.01 | * | 0.01 | ** |
|  | (0.00) |  | (0.00) |  | (0.00) |  | (0.00) |  | (0.00) |  | (0.00) |  |
| Constant | 1.78 | *** | 2.04 | *** | 2.12 | *** | 1.78 | *** | 2.04 | *** | 2.12 | *** |
|  | (0.14) |  | (0.09) |  | (0.13) |  | (0.14) |  | (0.09) |  | (0.13) |  |
| R-Squared | 0.02 |  | 0.04 |  | 0.05 |  | 0.02 |  | 0.04 |  | 0.05 |  |
| Observations | 40466 |  | 40466 |  | 40466 |  | 40466 |  | 40466 |  | 40466 |  |

Note: * 0.1 ** 0.05 *** 0.01.

**Table S4c. UQR coefficients for number of children across men’s hourly wage distribution, controlling for inequality levels. United Kingdom. Online Only.**

|  |  |  | M2: FE |  |  |  |  |  | M3: FEGS | |  |  |
| --- | --- | --- | --- | --- | --- | --- | --- | --- | --- | --- | --- | --- |
|  | Q20 |  | Q50 |  | Q80 |  | Q20 |  | Q50 |  | Q80 |  |
|  | b/se |  | b/se |  | b/se |  | b/se |  | b/se |  | b/se |  |
| Nr children | -0.03 | *** | 0.00 |  | 0.03 | ** | -0.04 | *** | 0.00 |  | 0.02 |  |
|  | (0.01) |  | (0.01) |  | (0.02) |  | (0.01) |  | (0.01) |  | (0.02) |  |
| Gini (std) | 0.03 | *** | 0.03 | *** | 0.03 | *** | 0.03 | *** | 0.03 | *** | 0.04 | *** |
|  | (0.01) |  | (0.01) |  | (0.01) |  | (0.01) |  | (0.01) |  | (0.01) |  |
| Nr children*Gini (std) | -0.01 | * | -0.01 | ** | 0.00 |  | -0.01 | * | -0.01 | ** | -0.00 |  |
|  | (0.01) |  | (0.01) |  | (0.01) |  | (0.01) |  | (0.01) |  | (0.01) |  |
| Ever parent x experience | | |  |  |  |  | 0.00 |  | 0.00 |  | 0.01 | * |
|  |  |  |  |  |  |  | (0.01) |  | (0.00) |  | (0.01) |  |
| Experience | 0.08 | *** | 0.05 | *** | 0.03 | ** | 0.08 | *** | 0.05 | *** | 0.02 |  |
|  | (0.01) |  | (0.01) |  | (0.01) |  | (0.01) |  | (0.01) |  | (0.01) |  |
| Experience sq | -0.00 | *** | -0.00 | *** | -0.00 | *** | -0.00 | *** | -0.00 | *** | -0.00 | *** |
|  | (0.00) |  | (0.00) |  | (0.00) |  | (0.00) |  | (0.00) |  | (0.00) |  |
| A-levels | 0.16 | * | -0.07 |  | -0.15 | ** | 0.15 | * | -0.07 |  | -0.15 | ** |
|  | (0.08) |  | (0.06) |  | (0.07) |  | (0.08) |  | (0.06) |  | (0.07) |  |
| Tertiary | 0.12 |  | 0.04 |  | -0.08 |  | 0.12 |  | 0.04 |  | -0.08 |  |
|  | (0.08) |  | (0.08) |  | (0.10) |  | (0.08) |  | (0.07) |  | (0.10) |  |
| Married | 0.07 | ** | 0.05 |  | -0.06 |  | 0.07 | ** | 0.05 | * | -0.05 |  |
|  | (0.04) |  | (0.03) |  | (0.04) |  | (0.04) |  | (0.03) |  | (0.04) |  |
| Cohabiting | 0.09 | *** | 0.03 |  | -0.05 | * | 0.09 | *** | 0.03 |  | -0.04 |  |
|  | (0.03) |  | (0.02) |  | (0.03) |  | (0.03) |  | (0.02) |  | (0.03) |  |
| Managers&Professionals | 0.02 |  | 0.07 | *** | 0.06 | ** | 0.02 |  | 0.07 | *** | 0.06 | ** |
|  | (0.03) |  | (0.02) |  | (0.02) |  | (0.03) |  | (0.02) |  | (0.02) |  |
| Technicians&Service &Clerks | -0.05 | * | -0.00 |  | -0.00 |  | -0.04 |  | -0.00 |  | 0.00 |  |
|  | (0.03) |  | (0.02) |  | (0.02) |  | (0.03) |  | (0.02) |  | (0.02) |  |
| Private Sector | -0.12 | *** | 0.02 |  | 0.10 | *** | -0.12 | *** | 0.02 |  | 0.10 | *** |
|  | (0.03) |  | (0.03) |  | (0.03) |  | (0.03) |  | (0.03) |  | (0.03) |  |
| GDP | 0.00 | *** | 0.00 | *** | 0.00 | *** | 0.00 | *** | 0.00 | *** | 0.00 | *** |
|  | (0.00) |  | (0.00) |  | (0.00) |  | (0.00) |  | (0.00) |  | (0.00) |  |
| Unemployment rate | -0.02 | *** | -0.01 | *** | -0.00 |  | -0.02 | *** | -0.01 | *** | -0.00 |  |
|  | (0.01) |  | (0.01) |  | (0.01) |  | (0.01) |  | (0.01) |  | (0.01) |  |
| Constant | 1.02 | *** | 1.67 | *** | 2.05 | *** | 1.01 | *** | 1.67 | *** | 2.03 | *** |
|  | (0.14) |  | (0.12) |  | (0.16) |  | (0.14) |  | (0.12) |  | (0.16) |  |
| R-Squared | 0.11 |  | 0.13 |  | 0.12 |  | 0.11 |  | 0.13 |  | 0.12 |  |
| Sample Size | 18455 |  | 18455 |  | 18455 |  | 18455 |  | 18455 |  | 18455 |  |

Note: * 0.1 ** 0.05 *** 0.01. Models also include region dummies.

**Table S5. UQR coefficients for number of children across men’s hourly wage distribution, pre and post 2007 reform. Germany. Online Only.**

|  |  |  |  |  | UQR-FEGS | |  |  |  |  |  |  |
| --- | --- | --- | --- | --- | --- | --- | --- | --- | --- | --- | --- | --- |
|  | 1995/2006 | | | | | | 2007/2016 | | | | | |
|  | Q20 |  | Q50 |  | Q80 |  | Q20 |  | Q50 |  | Q80 |  |
|  | b/se |  | b/se |  | b/se |  | b/se |  | b/se |  | b/se |  |
| Nr children | -0.02 |  | 0.00 |  | 0.02 |  | -0.02 |  | 0.01 |  | 0.04 | * |
|  | (0.01) |  | (0.01) |  | (0.01) |  | (0.02) |  | (0.01) |  | (0.02) |  |
| Ever parent X experience | -0.00 |  | -0.00 |  | -0.01 |  | 0.01 |  | -0.01 |  | -0.00 |  |
|  | (0.01) |  | (0.00) |  | (0.01) |  | (0.01) |  | (0.01) |  | (0.01) |  |
| Experience | 0.05 | *** | 0.04 | *** | 0.02 | * | -0.01 |  | -0.00 |  | 0.00 |  |
|  | (0.01) |  | (0.01) |  | (0.01) |  | (0.05) |  | (0.04) |  | (0.03) |  |
| Experience sq | -0.00 | *** | -0.00 | *** | -0.00 | *** | -0.00 | *** | -0.00 | ** | -0.00 | *** |
|  | (0.00) |  | (0.00) |  | (0.00) |  | (0.00) |  | (0.00) |  | (0.00) |  |
| No education, low sec | -0.04 |  | -0.01 |  | 0.03 |  | -0.13 |  | 0.19 | ** | 0.13 | *** |
|  | (0.04) |  | (0.03) |  | (0.04) |  | (0.25) |  | (0.08) |  | (0.04) |  |
| Abitur+vocational | 0.08 | * | 0.03 |  | 0.02 |  | 0.16 |  | 0.00 |  | -0.08 |  |
|  | (0.05) |  | (0.04) |  | (0.05) |  | (0.16) |  | (0.09) |  | (0.08) |  |
| Tertiary | 0.15 | ** | 0.10 | * | 0.04 |  | 0.44 | ** | 0.29 |  | 0.05 |  |
|  | (0.07) |  | (0.05) |  | (0.07) |  | (0.20) |  | (0.19) |  | (0.11) |  |
| Married | 0.05 |  | 0.04 |  | -0.02 |  | 0.14 | *** | 0.02 |  | -0.01 |  |
|  | (0.03) |  | (0.02) |  | (0.03) |  | (0.04) |  | (0.03) |  | (0.05) |  |
| Cohabiting | 0.03 |  | 0.01 |  | -0.03 |  | 0.08 | ** | 0.02 |  | -0.03 |  |
|  | (0.03) |  | (0.02) |  | (0.03) |  | (0.04) |  | (0.02) |  | (0.03) |  |
| Managers&Professionals | -0.05 | * | 0.02 |  | 0.03 |  | -0.05 |  | 0.01 |  | -0.00 |  |
|  | (0.03) |  | (0.02) |  | (0.03) |  | (0.04) |  | (0.03) |  | (0.03) |  |
| Technicians&Service&Clerks | -0.08 | *** | -0.03 |  | -0.04 |  | -0.04 |  | 0.02 |  | -0.02 |  |
|  | (0.03) |  | (0.02) |  | (0.02) |  | (0.04) |  | (0.02) |  | (0.02) |  |
| East Germany | -0.36 | *** | -0.05 |  | 0.06 |  | -0.25 | *** | -0.14 |  | -0.06 |  |
|  | (0.10) |  | (0.06) |  | (0.05) |  | (0.09) |  | (0.09) |  | (0.06) |  |
| Constant | 1.93 | *** | 2.23 | *** | 2.80 | *** | 2.89 | *** | 3.21 | *** | 3.67 | *** |
|  | (0.11) |  | (0.07) |  | (0.12) |  | (1.00) |  | (0.98) |  | (0.63) |  |
|  |  |  |  |  |  |  |  |  |  |  |  |  |
| R-Squared | 0.017 |  | 0.04 |  | 0.05 |  | 0.036 |  | 0.042 |  | 0.034 |  |
| Sample Size | 21603 |  | 21603 |  | 21603 |  | 18863 |  | 18863 |  | 18863 |  |

Note: * 0.1 ** 0.05 *** 0.01. Models also include year dummies.
